# The TAL Effector AvrBs3 from *Xanthomonas campestris* pv. *vesicatoria* Contains Multiple Export Signals and Can Enter Plant Cells in the Absence of the Type III Secretion Translocon

**DOI:** 10.3389/fmicb.2017.02180

**Published:** 2017-11-09

**Authors:** Felix Scheibner, Sylvestre Marillonnet, Daniela Büttner

**Affiliations:** ^1^Institute of Biology, Department of Genetics, Martin Luther University Halle-Wittenberg, Halle, Germany; ^2^Leibniz Institute of Plant Biochemistry, Halle, Germany

**Keywords:** transcription activator-like effector, AvrBs3, *Xanthomonas*, type III secretion, chaperone, translocon

## Abstract

Pathogenicity of the Gram-negative plant-pathogenic bacterium *Xanthomonas campestris* pv. *vesicatoria* depends on a type III secretion (T3S) system which translocates effector proteins into plant cells. Effector protein delivery is controlled by the T3S chaperone HpaB, which presumably escorts effector proteins to the secretion apparatus. One intensively studied effector is the transcription activator-like (TAL) effector AvrBs3, which binds to promoter sequences of plant target genes and activates plant gene expression. It was previously reported that type III-dependent delivery of AvrBs3 depends on the N-terminal protein region. The signals that control T3S and translocation of AvrBs3, however, have not yet been characterized. In the present study, we show that T3S and translocation of AvrBs3 depend on the N-terminal 10 and 50 amino acids, respectively. Furthermore, we provide experimental evidence that additional signals in the N-terminal 30 amino acids and the region between amino acids 64 and 152 promote translocation of AvrBs3 in the absence of HpaB. Unexpectedly, *in vivo* translocation assays revealed that AvrBs3 is delivered into plant cells even in the absence of HrpF, which is the predicted channel-forming component of the T3S translocon in the plant plasma membrane. The presence of HpaB- and HrpF-independent transport routes suggests that the delivery of AvrBs3 is initiated during early stages of the infection process, presumably before the activation of HpaB or the insertion of the translocon into the plant plasma membrane.

## Introduction

Many Gram-negative bacterial pathogens translocate effector proteins into eukaryotic host cells to modulate host cellular pathways such as defense responses to their own benefit (Raymond et al., 2013; Ashida et al., 2015; Santos and Finlay, 2015; Büttner, 2016; Ensminger, 2016; Grabowski et al., 2017). Effector protein translocation often depends on the type III secretion (T3S) system, which is an essential pathogenicity factor of many bacterial species and is related to the bacterial flagellum (Büttner, 2012; Diepold and Armitage, 2015). At least eight components of T3S systems are conserved in different bacterial species and were designated Sct (secretion and cellular translocation) proteins in animal-pathogenic bacteria followed by a letter, which indicates the corresponding T3S system components from *Yersinia* species (Hueck, 1998; Deng et al., 2017). Sct proteins are mainly involved in the assembly of the membrane-spanning part of the secretion apparatus, which consists of ring structures in the inner and outer bacterial membrane and a predicted periplasmic inner rod (Büttner, 2012). The inner membrane rings associate with the export apparatus, which is assembled by five transmembrane proteins and presumably forms a transport channel for secreted proteins (Diepold et al., 2011; Büttner, 2012; Dietsche et al., 2016; Deng et al., 2017). Components of the export apparatus are connected with the cytoplasmic ATPase complex, which provides the energy for secretion and/or unfolds secreted proteins during transport (Büttner, 2012; Deng et al., 2017). The ATPase presumably interacts with members of the SctQ family, which assemble as cytoplasmic ring or pod-like structures and are potential docking sites for T3S substrates (Büttner, 2012; Deng et al., 2017; Hu et al., 2017).

Extracellular components of translocation-associated T3S systems from animal-pathogenic bacteria include the T3S needle, which serves as a conduit for T3S substrates to the host-pathogen interface. T3S needles are 40–80 nm long and thus significantly shorter than the T3S pilus from plant-pathogenic bacteria, which reaches a length of up to 2 μm and spans the plant cell wall (Büttner, 2012). Needle and pilus are directly or indirectly connected to the T3S translocon, which inserts as a channel-like complex into the eukaryotic plasma membrane and mediates effector protein translocation (Mueller et al., 2008; Mattei et al., 2011; Galan et al., 2014). T3S translocons from animal-pathogenic bacteria often consist of two conserved hydrophobic translocators, which form the transmembrane channel, and a third hydrophilic translocator, which presumably provides an assembly platform for the translocon channel at the tip of the needle (Mattei et al., 2011). Notably, translocon-dependent protein delivery into eukaryotic cells does not appear to be restricted to proteins that travel inside the T3S system but was also reported for external proteins. Thus, the effector protein YopH from *Yersinia* spp. is delivered by the T3S translocon when present on the bacterial surface (Akopyan et al., 2011). Similarly, translocon-dependent delivery was reported for the type V-secreted autotransporter EspC from enteropathogenic *Escherichia coli*, which interacts with translocon proteins, suggesting that the T3S translocon also transports T3S-unrelated proteins (Vidal and Navarro-Garcia, 2008; Tejeda-Dominguez et al., 2017). The translocon is usually essential for type III-dependent effector protein delivery, however, several type III effector proteins from animal-pathogenic bacteria including SspH1 from *Salmonella* spp. and YopM from *Yersinia* spp. can also enter eukaryotic cells independently of the translocon, presumably via endocytosis or direct transport across the plasma membrane (Rüter et al., 2010; Scharnert et al., 2013; Lubos et al., 2014). A similar mechanism has not yet been reported for effector proteins from plant-pathogenic bacteria.

In contrast to T3S translocons from animal-pathogenic bacteria, the predicted translocon from plant-pathogenic bacteria is less well conserved among different species and the mechanisms underlying effector protein translocation are not yet understood. In *Xanthomonas campestris* pv. *vesicatoria* (also designated *Xanthomonas euvesicatoria*), the secreted HrpF protein was identified as putative channel-forming translocon protein (Büttner et al., 2002). Mutant studies and *in vivo* translocation assays suggest that HrpF is essential for pathogenicity of *X. campestris* pv. *vesicatoria* and effector protein translocation (Büttner et al., 2002; Hotson et al., 2003; Jiang et al., 2009; Teper et al., 2016). Homologous proteins are present in many *Xanthomonas* species and *Ralstonia solanacearum*. Furthermore, HrpF shares limited sequence similarity with the putative translocon protein HrpK1 from *Pseudomonas syringae* (Kvitko et al., 2007). HrpK1 contributes to but is not essential for pathogenicity of *P. syringae* as well as for efficient effector protein delivery and HR induction on tobacco plants (Petnicki-Ocwieja et al., 2005; Kvitko et al., 2007). Accessory hydrophilic proteins, designated harpins, presumably contribute to effector protein translocation in *P. syringae* and were also identified in other bacterial species (Kvitko et al., 2007; Choi et al., 2013; Ji and Dong, 2015).

*X. campestris* pv. *vesicatoria*, which causes bacterial spot disease in pepper and tomato plants, is one of the model systems for the analysis of T3S systems in plant-pathogenic bacteria (Jones et al., 2004; Potnis et al., 2015). The T3S system from *X. campestris* pv. *vesicatoria* is essential for pathogenicity and is encoded by the chromosomal *hrp* (hypersensitive response and pathogenicity) gene cluster (Büttner and Bonas, 2002). Eleven *hrp* gene products, referred to as Hrc (Hrp conserved), are conserved in animal- and/or plant-pathogenic bacteria and presumably constitute the core components of the secretion apparatus (Büttner and Bonas, 2002). Additional components are encoded by non-conserved *hrp* genes and include for instance the predicted inner rod proteins HrpB1 and HrpB2, which presumably form an assembly platform for the T3S pilus, the pilus protein HrpE and the translocon protein HrpF (Büttner et al., 2002; Weber et al., 2005; Hartmann et al., 2012; Hausner et al., 2013). An additional protein, which might contribute to effector delivery, is the secreted XopA protein, which is encoded in the flanking region of the *hrp* gene cluster (Noël et al., 2002).

The *hrp* gene cluster also encodes Hpa (Hrp-associated) proteins which contribute to but are not essential for type III-dependent effector protein translocation. One example is the general T3S chaperone HpaB, which is essential for pathogenicity and promotes effector protein translocation (Büttner et al., 2004). HpaB presumably targets effectors to the ATPase HrcN of the T3S system and is involved in the recognition of translocation signals (Büttner et al., 2006; Lorenz and Büttner, 2009; Scheibner et al., 2017). The activity of HpaB is controlled by its secreted regulator HpaA, which interacts with and likely inhibits HpaB. Secretion of HpaA presumably liberates HpaB and thus activates effector protein secretion (Lorenz et al., 2008).

The T3S system from *X. campestris* pv. *vesicatoria* translocates more than 30 effector proteins into plant cells (Büttner et al., 2006, 2007; Szczesny et al., 2010a; Schulze et al., 2012). In many cases, the precise biochemical functions and plant targets of effectors are still unknown (Büttner and Bonas, 2010). One of the best studied type III effectors from *X. campestris* pv. *vesicatoria* is AvrBs3, which is a member of the transcription activator-like (TAL) effector family and acts as a transcription factor in plant cells (Boch and Bonas, 2010). TAL effectors contain C-terminal nuclear localization signals (NLSs) and are imported into the plant cell nucleus (Boch and Bonas, 2010). The central region of TAL effectors consists of a variable number of amino acid repeats with a predominant length of 34 amino acids and mediates DNA binding (Boch and Bonas, 2010). The repeats are almost sequence-identical with the exception of two variable residues (RVDs, repeat variable diresidues) at amino acid positions 12 and 13 of each repeat (Boch and Bonas, 2010). The RVDs allow the base-specific binding of TAL effectors to sequences in the promoter regions of plant target genes (Boch et al., 2009; Moscou and Bogdanove, 2009; Deng et al., 2012; Mak et al., 2012). The subsequent modulation of plant gene expression by TAL effectors depends on their C-terminal acidic activation domain (AAD) (Boch and Bonas, 2010). Among the plant target genes is the *Bs3* resistance gene, which is present in AvrBs3-responsive pepper plants and encodes a flavin monooxygenase. Bs3 initiates the induction of a hypersensitive response (HR), which is a local rapid cell death at the infection site and presumably restricts bacterial multiplication (Römer et al., 2009; Wu et al., 2014).

Delivery of AvrBs3 by the T3S system depends on the N-terminal 152 amino acids which presumably contain the export signal (Szurek et al., 2002; Noël et al., 2003). We previously reported that the N-terminal 50 amino acids of AvrBs3 are sufficient for T3S (Büttner et al., 2004). Yet, the precise location of T3S and translocation signals in AvrBs3 has not been determined. Secretion and translocation signals are usually located in the N-terminal regions of T3S substrates and are not conserved on the amino acid level (Sory et al., 1995; Schesser et al., 1996; Rüssmann et al., 2002; Schechter et al., 2004; Arnold et al., 2009). However, they often contain specific amino acid compositions or patterns such as a high content of polar amino acids as was reported for effector proteins from the plant-pathogenic bacterium *P. syringae* (Guttman et al., 2002; Petnicki-Ocwieja et al., 2002; Greenberg and Vinatzer, 2003; Schechter et al., 2004, 2006; Arnold et al., 2009; Löwer and Schneider, 2009; Samudrala et al., 2009; Buchko et al., 2010). Evidence for an essential role of characteristic amino acids for T3S or translocation, however, is still missing (Schechter et al., 2012).

In the present study, we localized T3S and translocation signals in the TAL effector AvrBs3. The results of *in vitro* T3S and *in vivo* translocation assays revealed that AvrBs3 contains separate signals, which control T3S and translocation in the presence or absence of the T3S chaperone HpaB. Furthermore, the analysis of AvrBs3 translocation by HR- and fluorescence based translocation assays showed that AvrBs3 enters plant cells in the absence of a functional translocon, suggesting that it is translocated during early stages of the infection process prior to the assembly of the translocon.

## Materials and methods

### Bacterial strains and growth conditions

Bacterial strains and plasmids used in this study are listed in Table 1. *Escherichia coli* strains were cultivated at 37°C in lysogeny broth (LB) medium and *X. campestris* pv. *vesicatoria* strains at 30°C in nutrient-yeast extract-glycerol (NYG) medium (Daniels et al., 1984) or minimal medium A (Ausubel et al., 1996) at pH 7.0 supplemented with 10 mM sucrose and 0.3% casamino acids.

**Table 1:** Bacterial strains and plasmids used in this study.

**Strain/plasmid**	**Relevant characteristics^a^**	**References or Sources**
***X. campestris*** **pv**. ***vesicatoria***		
85-10	Pepper-race 2; wild type; Rif^r^	Canteros, 1990; Kousik and Ritchie, 1998
85-10Δ*hpaB*	Derivative of strain 85-10 deleted in *hpaB*	Büttner et al., 2004
85-10Δ*hrpE*	Derivative of strain 85-10 deleted in *hrpE*	Weber et al., 2005
85-10Δ*hrpF*	Derivative of strain 85-10 deleted in *hrpF*	Büttner et al., 2002
85-10Δ*hrpF*Δ*xopA*	Derivative of strain 85-10 deleted in *hrpF* and *xopA*	This study
85-10Δ*xpsDΔxcsD*	Derivative of strain 85-10 deleted in the secretin-encoding genes *xpsD* and *xcsD* of the Xcs- and Xps-T2S gene clusters	Szczesny et al., 2010b
85-10Δ*hrpF*Δ*xpsDΔxcsD*	Derivative of strain 85-10 lacking *hrpF* and the secretin genes *xcsD* and *xpsD*	This study
85^*^	85-10 derivative containing the hrpG^*^ mutation	Wengelnik et al., 1999
85^*^Δ*hpaB*	Derivative of strain 85^*^ deleted in *hpaB*	Büttner et al., 2004
85^*^Δ*hrpF*	Derivative of strain 85^*^ deleted in *hrpF*	Büttner et al., 2002
85^*^Δ*hpaBΔhrpF*	Derivative of strain 85^*^ deleted in *hpaB* and *hrpF*	Büttner et al., 2004
85^*^Δ*hrcV*Δ*hpaB*	Derivative of strain 85^*^ deleted in *hrcV* and *hpaB*	Büttner et al., 2004
85^*^Δ*hrpFΔxopA*	Derivative of strain 85^*^ deleted in *hrpF* and *xopA*	This study
85^*^Δ*hrpE*	Derivative of strain 85^*^ deleted in *hrpE*	Weber et al., 2005
85^*^Δ*hrcN*	Derivative of strain 85^*^ deleted in *hrcN*	Lorenz and Büttner, 2009
82-8	Pepper-race 1; wild type; Rif^r^	Canteros, 1990; Kousik and Ritchie, 1998
82-8Δ*hrpF*	Derivative of strain 82-8 deleted in *hrpF*	This study
82-8Δ*hrpFΔxopA*	Derivative of strain 82-8 deleted in *hrpF* and *xopA*	This study
82-8Δ*hrcV*	Derivative of strain 82-8 deleted in *hrcV*	Kindly provided by U. Bonas
82^*^	82-8 derivative containing the hrpG^*^ mutation	Wengelnik et al., 1999
82^*^Δ*hrpF*	Derivative of strain 82^*^ deleted in *hrpF*	Büttner et al., 2002
82^*^Δ*hrcN*	Derivative of strain 82^*^ deleted in *hrcN*	Wengelnik et al., 1999
82^*^Δ*hpaB*	Derivative of strain 82^*^ deleted in *hpaB*	Büttner et al., 2004
***A. tumefaciens***		
GV2260	Contains Ti plasmid pGV2260, Rif^r^, Ap^r^	Deblaere et al., 1985
***E. coli***		
DH5λpir	F^−^*recA hsdR17(${\text{r}}_{k}^{-}$,${\text{m}}_{k}^{+}$) Φ80dlacZ DM15 [*λ*pir]*	Ménard et al., 1993
OneShot®TOP10	F^−^*mcrA* Δ(*mrr-hsdRMS-mcrBC*) ϕ80*lacZ*ΔM15 Δ*lacX74 recA1 ara*Δ*139* Δ(*ara-leu*)*7697 galU galK rpsL endA1 nupG* (Str^R^)	Invitrogen
**Plasmids**		
pRK2013	ColE1 replicon, TraRK^+^ Mob^+^; Km^r^	Figurski and Helinski, 1979
pUC57ΔBsaI	Derivative of pUC57 with mutated *Bsa*I site	Morbitzer et al., 2011
pBBR1MCS-5	Broad-host-range vector; *lac* promoter; Gm^r^	Kovach et al., 1995
pBRM	Golden Gate-compatible derivative of pBBR1MCS-5	Szczesny et al., 2010b
pBRM-P	Derivative of pBRM lacking the *lac* promoter	Szczesny et al., 2010b
pBR356	Derivative of pBBR1MCS-5 containing *avrBs3Δ2* downstream of the *lac* promoter and the *lacZ*α fragment, which is flanked by *Bsa*I sites	Scheibner et al., 2016
pBRMavrBs3	Derivative of pBRM encoding AvrBs3	Kindly provided by U. Bonas
pBRMavrBs3_1−10_-356	Derivative of pBR356 encoding AvrBs3_1−10_-AvrBs3Δ2	This study
pBRMavrBs3_1−20_-356	Derivative of pBR356 encoding AvrBs3_1−20_-AvrBs3Δ2	This study
pBRMavrBs3_1−30_-356	Derivative of pBR356 encoding AvrBs3_1−30_-AvrBs3Δ2	This study
pBRMavrBs3_1−40_-356	Derivative of pBR356 encoding AvrBs3_1−40_-AvrBs3Δ2	This study
pBRMavrBs3_1−50_-356	Derivative of pBR356 encoding AvrBs3_1−50_-AvrBs3Δ2	This study
pBRM-PxopJ_1−155_-356	Derivative of pBRM-P encoding XopJ_1−155_-AvrBs3Δ2	This study
pBRM-PxopJ_1−155_-avrBs3ΔN63	Derivative of pBRM-P encoding XopJ_1−155_-AvrBs3ΔN63	This study
pBRMavrBs3_1−10_-avrBs3ΔN63	Derivative of pBR356 encoding AvrBs3_1−10_-AvrBs3ΔN63	This study
pBRMavrBs3_1−30_-avrBs3ΔN63	Derivative of pBR356 encoding AvrBs3_1−30_-AvrBs3ΔN63	This study
pBRMavrBs3ΔN63	Derivative of pBRM encoding AvrBs3ΔN63 deleted in amino acids 2 - 63	This study
pGGA3	Golden Gate-compatible binary vector; contains backbone of pBGWFS7 and the *35S* promoter, allows the expression of genes in fusion with a C-terminal FLAG epitope-encoding sequence, Sm^r^	Kindly provided by U. Bonas; Karimi et al., 2005
pGGA3avrBs3	Derivative of pGGA3 encoding AvrBs3	This study
pGGA3avrBs3_1−10_-356	Derivative of pGGA3 containing *avrBs3_*1−10*_-avrBs3Δ2*	This study
pGGA3avrBs3_1−20_-356	Derivative of pGGA3 containing *avrBs3_*1−20*_-avrBs3Δ2*	This study
pICH77739	Derivative of pBIN19, *RK2* ori, contains *lacZ*a flanked by *Bpi*I sites; Km^r^	Weber et al., 2011a
pICH77739_dTALE-2	Derivative of pICH77739 encoding dTALE-2	Scheibner et al., 2016
pICH77739_dTALE-2ΔN	Derivative of pICH77739 encoding dTALE-2ΔN which is deleted in amino acids 2–64	Scheibner et al., 2016
pICH77739_AvrBs3_1−30_-dTALE-2ΔN	Derivative of pICH77739 encoding AvrBs3_1−30_-dTALE-2ΔN	This study
pICH77739_AvrBs3_1−50_-dTALE-2ΔN	Derivative of pICH77739 encoding AvrBs3_1−50_-dTALE-2ΔN	This study
pOK1	Suicide vector; *sacB sacQ mobRK2 oriR6K*; Sm^r^	Huguet et al., 1998
pOKΔhrpF	2-kb fragment containing the flanking regions of *hrpF* in pOK1	Büttner et al., 2002
pOxopA	Derivative of pOK1, contains the flanking regions of *xopA*	Noël et al., 2002

a *Ap, ampicillin; Km, kanamycin; Rif, rifampicin; Sm, spectinomycin; Str, streptomycin; Tc, tetracycline; r, resistant*.

### Plant material and plant inoculations

*X. campestris* pv. *vesicatoria* strains were inoculated into leaves of the near-isogenic pepper cultivars Early Cal Wonder (ECW), ECW-10R and ECW-30R, and *Bs3-* or *gfp-*transgenic *Nicotiana benthamiana* plants at concentrations of 4 × 10^8^ colony-forming units (CFU) ml^−1^ in 1 mM MgCl_2_ if not stated otherwise (Minsavage et al., 1990; Bonas et al., 1991; Kousik and Ritchie, 1998; Scheibner et al., 2016). *Agrobacterium tumefaciens* strains were infiltrated in 1 mM MgCl_2_ at a density of 8 × 10^8^ CFU ml^−1^. Infected pepper plants were incubated for 16 h of light at 28°C and 65% humidity, and 8 h of darkness at 22°C and 65% humidity. *N. benthamiana* plants were incubated for 16 h of light at 20°C and 75% humidity, and 8 h of darkness at 18°C and 70% humidity. The appearance of plant reactions was scored over a period of one to 12 dpi. For the better visualization of the HR, leaves were destained in 70% ethanol. Experiments were repeated at least twice; representative plant reactions are shown.

### Generation of expression constructs

For the generation of expression constructs encoding AvrBs3Δ2 fusion proteins under control of the *lac* promoter, *avrBs3* gene fragments were amplified by PCR from *X. campestris* pv. *vesicatoria* and cloned into the *Bsa*I sites of plasmid pBR356, which contains the *avrBs3*Δ*2* reporter gene (Scheibner et al., 2016). For the generation of constructs encoding AvrBs3Δ63 or AvrBs3Δ63 fusion proteins, modules encoding N-terminal, central and C-terminal regions of AvrBs3Δ63 and the respective fusion partners were cloned into the *Bsa*I sites of plasmid pBRM. The stop codon of *avrBs3* was included in these constructs. *xopJ*_*1-155*_*-avrBs3*Δ*2* and *xopJ*_*1-155*_*-avrBs3*Δ*63* were expressed under control of the native *xopJ* promoter in plasmid pBRM-P. For this, fragments containing the first 155 codons of *xopJ* and 720 bp of the upstream region including the *xopJ* promoter (Noël et al., 2003) were ligated with fragments encoding the N-terminal, central and C-terminal regions of AvrBs3Δ63.

For the generation of binary constructs for *A. tumefaciens-*mediated transient gene expression, *avrBs3*_*1-10*_ and *avrBs3*_*1-20*_ were amplified by PCR and ligated with modules encoding N-terminal, central and C-terminal regions of AvrBs3Δ2 into the *Bsa*I sites of the binary vector pGGA3 downstream of the *35S* promoter. Similarly, modules encoding N-terminal, central and C-terminal regions of AvrBs3 were cloned into pGGA3. The module encoding the C-terminal region of AvrBs3 lacked the native stop codon and was therefore expressed in fusion with a FLAG epitope-encoding sequence provided by vector pGGA3.

Expression constructs encoding dTALE-2 and derivatives thereof were generated by Golden-Gate assembly of individual DNA modules encoding N-terminal, central and C-terminal regions of dTALE-2, dTALE-2ΔN as well as N-terminal regions of the respective fusion partners. All primers used in this study are listed in Table 2. Plasmids were introduced into *X. campestris* pv. *vesicatoria* by electroporation or by conjugation using pRK2013 as a helper plasmid in triparental matings (Figurski and Helinski, 1979).

**Table 2:** Primers used in this study.

**Primer**	**Sequence (5′-3′)^a^**
***avrBs3*** **EXPRESSION CONSTRUCTS**	
avrBs3 Bsa for	TTT GGTCTC T *TATG* GATCCCATTCGTTC
avrBs3 Bsa 10 for	*TATG*GATCCCATTCGTTCGCGCACACCAAGT
avrBs3 Bsa 10 rev	*GATC*ACTTGGTGTGCGCGAACGAATGGGATC
avrBs3 Bsa 20 rev	TTT GGTCTC T *GATC* TTGGGGTCCGGGCAGAAG
avrBs3 Bsa 30 rev	TTT GGTCTC T *GATC* ACGATCTGCAGTC
avrBs3 Bsa 50 rev	TTT GGTCTC T *GATC* GGACATCGTCCGCCGAGC
xopJ prom720 for	TTT GGTCTC T *ATTC* CTGTATCTGTGCATCGTATTG
xopJ Bsa 155 rev	TTT GGTCTC T *CACC* TGACTGGCGATCAGAGATAGC
***dTALE-2*** **EXPRESSION CONSTRUCTS**	
avrBs3 AATG Bpi for	TTT GAAGAC AA *AATG* GATCCCATTCGTTC
avrBs3 CTGA Bpi 30 rev	TTT GAAGAC AA *CTGA* ACGATCTGCAGTCGGCTG
avrBs3 CTGA Bpi 50 rev	TTT GAAGAC AA *CTGA* GGACATCGTCCGCCGAGC
**RT-PCR PRIMER**	
RT-Bs3-F	GTAACTTCTTGGTTAATGGAGAGTGAATTG
RT-Bs3-R	TGATTCTTGTGCTACATTTGTTCTTTCC
Elo-F/Aso	AGTCAACTACCACTGGTCAC
Elo-R/Aso	GTGCAGTAGTACTTAGTGGTC

a *Overhangs generated by BsaI or BpiI digestion are written in italics*.

### Generation of *X. campestris* pv. *vesicatoria* deletion mutants

For the generation of *X. campestris* pv. *vesicatoria* deletion mutants (see Table 1), derivatives of the suicide vector pOK1, which contained the flanking regions of the deleted genes, were transferred to *X. campestris* pv. *vesicatoria* recipient strains by triparental conjugation. Double crossovers resulted in deletion mutants which were selected as described previously (Huguet et al., 1998).

### Analysis of protein extracts and *in Vitro* secretion assays

For protein analysis, bacteria were cultivated over-night in liquid NYG medium and cells were harvested by centrifugation. Equal amounts of proteins adjusted according to the optical densities of the cultures were analyzed by immunoblotting, using AvrBs3- or HrpF-specific antibodies. For the analysis of *in vitro* T3S, bacteria were incubated in MA medium at pH 5.3 for 1.5 h in the presence of thiamine and bovine serum albumin as described previously (Rossier et al., 1999). Secreted proteins were separated from bacterial cells by filtration and precipitated by trichloroacetic acid. Equal amounts of bacterial total cell extracts and culture supernatants (adjusted according to the optical densities of the cultures) were analyzed by SDS-PAGE and immunoblotting using antibodies specific for AvrBs3, the translocon protein HrpF, the inner membrane ring protein HrcJ and the predicted periplasmic inner rod protein HrpB1, respectively (Knoop et al., 1991; Rossier et al., 2000; Büttner et al., 2002). Horseradish peroxidase-labeled anti-rabbit antibodies (GE Healthcare) were used as secondary antibodies. Experiments were repeated twice.

### RNA analysis

For transcript analysis via semiquantitative reverse-transcription PCR (RT-PCR), strain 85^*^ and derivatives thereof ectopically expressing *avrBs3* were inoculated at concentrations of 4 × 10^8^ CFU ml^−1^ and derivatives of strain 82-8 at concentrations of 8 × 10^8^ CFU ml^−1^ into leaves of ECW-30R pepper plants or *Bs3*-transgenic *N. benthamiana* plants. 16 leaf discs (2 mm diameter) were harvested 8 h (for derivatives of strain 85^*^) or 24 h (for derivatives of strain 82-8) post infiltration, pooled and immediately frozen in liquid nitrogen. RNA was isolated with the QIAGEN RNeasy Plant Mini Kit, and samples were treated with DNase I (Roche) for 30 min. cDNA was synthesized from 2 μg of total RNA with the RevertAid H Minus First Strand cDNA synthesis kit (Thermo Scientific) using random hexamer primers. Four microlitres of a 1:50 diluted cDNA solution were used as template in an RT-PCR with up to 40 cycles of denaturation, annealing and elongation. The constitutively expressed gene *Elongation factor 1*α (*EF1*α) was amplified as control (Römer et al., 2007). To exclude contaminations by genomic DNA, all reactions were also performed in the absence of reverse transcriptase. The experiments were repeated at least twice with similar results.

## Results

### The N-terminal 10 amino acids of AvrBs3 contain a T3S signal

**Figure 1:**
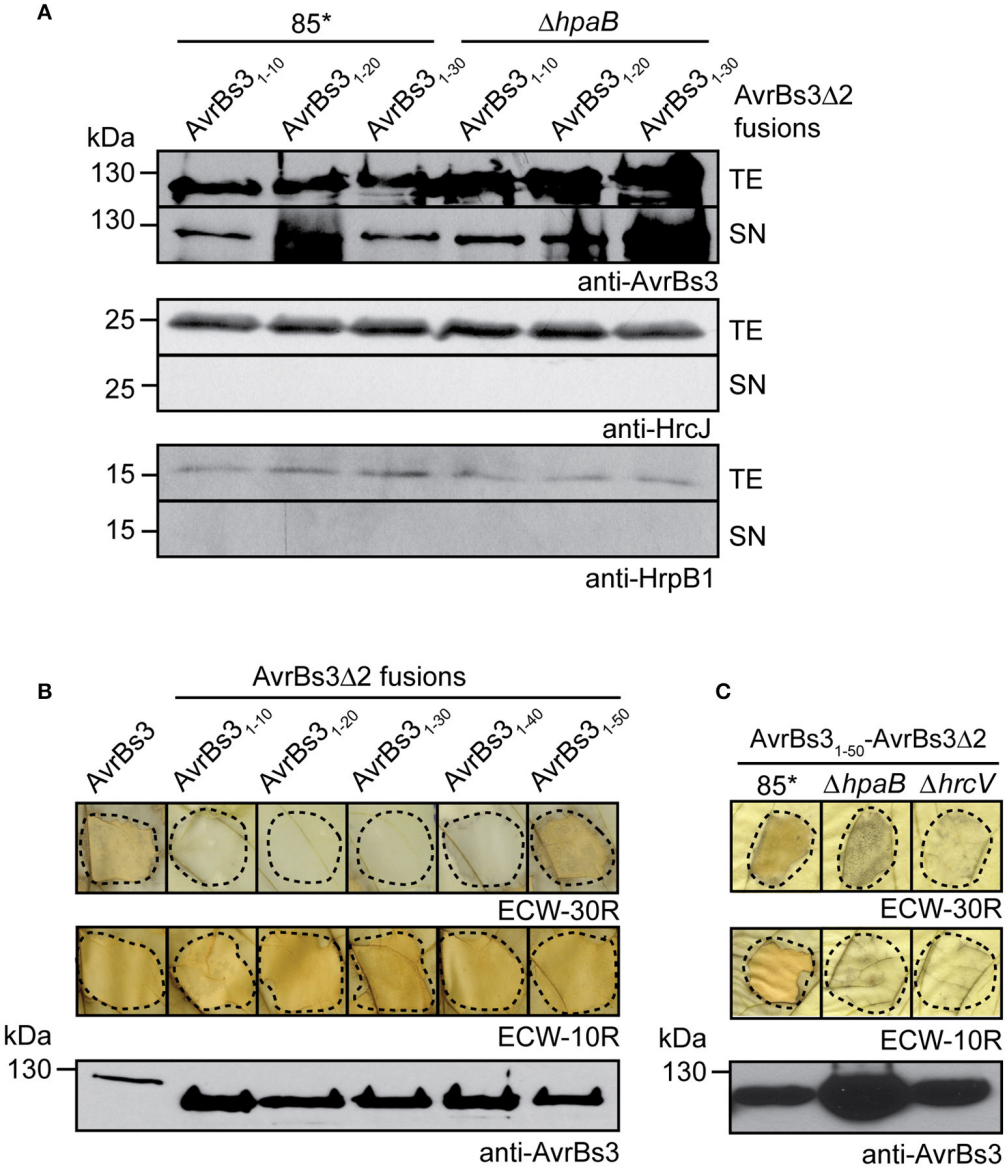
Localization of T3S and translocation signals in AvrBs3. **(A)** The N-terminal 10 amino acids of AvrBs3 target AvrBs3Δ2 for secretion in wild-type and *hpaB* deletion mutant strains. *X. campestris* pv. *vesicatoria* strains 85^*^ and 85^*^Δ*hpaB* (Δ*hpaB*) ectopically expressing *avrBs3*Δ*2* fusions as indicated were incubated in secretion medium. Total cell extracts (TE) and culture supernatants (SN) were analyzed by immunoblotting, using AvrBs3-specific antibodies. All AvrBs3Δ2 fusions were reproducibly secreted by both strains, the relative secretion levels, however, varied in different experiments. As control, the blots were reprobed with antibodies against the inner membrane protein HrcJ and the periplasmic predicted inner rod protein HrpB1. **(B)** The N-terminal 50 amino acids of AvrBs3 contain the translocation signal. Strain 85^*^ ectopically expressing *avrBs3* or *avrBs3*Δ*2* fusions as indicated was infiltrated into leaves of AvrBs3-responsive ECW-30R and AvrBs1-responsive ECW-10R pepper plants. For the better visualization of the HR, leaves were destained in ethanol 3 dpi. Dashed lines indicate the infiltrated areas. Equal amounts of cell extracts were analyzed by immunoblotting using an AvrBs3-specific antiserum. **(C)** Translocation of AvrBs3_1−50_-AvrBs3Δ2 depends on the T3S system. Strains 85^*^, 85^*^Δ*hpaB* (Δ*hpaB*) and the T3S-deficient strain 85^*^Δ*hrcV* (Δ*hrcV*) ectopically expressing *avrBs3*_1−50-_*avrBs3*Δ*2* were infiltrated into leaves of AvrBs3-responsive ECW-30R and AvrBs1-responsive ECW-10R pepper plants. Plant reactions and protein synthesis were analyzed as described in **(B)**.

To localize the T3S signal in AvrBs3, we generated fusion proteins consisting of the first 10, 20, or 30 amino acids, respectively, of AvrBs3 and AvrBs3Δ2, which is an N-terminal deletion derivative of AvrBs3. AvrBs3Δ2 lacks amino acids 2–152 and thus the T3S and translocation signal. However, AvrBs3Δ2 contains the effector domain and induces the HR in AvrBs3-responsive plants when delivered as fusion partner of a functional translocation signal (Szurek et al., 2002; Noël et al., 2003; Drehkopf et al., 2017). AvrBs3Δ2 fusion proteins were analyzed in *X. campestris* pv. *vesicatoria* strain 85^*^, which is a derivative of the wild-type strain 85-10 and contains HrpG^*^, a constitutively active version of the *hrp* gene regulator HrpG (Rossier et al., 1999; Wengelnik et al., 1999). When bacteria were cultivated in secretion medium, AvrBs3_1−10_-, AvrBs3_1−20_- and AvrBs3_1−30_-AvrBs3Δ2 fusion proteins were detected in the culture supernatants (Figure 1A). Similar results were obtained for strain 85^*^Δ*hpaB*, which lacks the T3S chaperone HpaB (Figure 1A; Büttner et al., 2004). As controls, we analyzed the inner membrane-associated protein HrcJ and the predicted periplasmic inner rod protein HrpB1. Both proteins were only detected in cell extracts of strains 85^*^ and 85^*^Δ*hpaB*, suggesting that no cell lysis had occurred (Figure 1A). We conclude from these data that the N-terminal 10–30 amino acids of AvrBs3 target AvrBs3Δ2 for secretion, even in the absence of the T3S chaperone HpaB. This is in contrast to the full-length AvrBs3 protein, which depends on HpaB for efficient secretion (Büttner et al., 2004).

### The N-terminal 50 amino acids of AvrBs3 contain a translocation signal

For the analysis of translocation signals in AvrBs3, we performed *in vivo* translocation assays with AvrBs3Δ2 fusion proteins. For this, strain 85^*^ ectopically expressing individual *avrBs3*Δ*2* fusions was infiltrated into leaves of AvrBs3-responsive ECW-30R pepper plants. AvrBs3_1−50_-AvrBs3Δ2 induced the HR in AvrBs3-responsive ECW-30R pepper plants when delivered by strain 85^*^, suggesting that the N-terminal 50 amino acids contain a functional translocation signal (Figure 1B). HR induction by AvrBs3_1−50_-AvrBs3Δ2 was dependent on the T3S system because it was macroscopically not detectable when AvrBs3_1−50_-AvrBs3Δ2 was analyzed in the T3S-deficient strain 85^*^Δ*hrcV* (Figure 1C). In contrast to AvrBs3_1−50_-AvrBs3Δ2, AvrBs3Δ2 fusions containing the N-terminal 10, 20, 30, or 40 amino acids of AvrBs3 did not induce a visible HR when analyzed in strain 85^*^, suggesting that they were not or not efficiently translocated (Figure 1B). Immunoblot analysis of bacterial cell extracts revealed that all fusion proteins were stably synthesized (Figure 1B).

As control, bacteria were infiltrated into leaves of ECW-10R pepper plants, which contain the resistance gene *Bs1* and initiate the HR upon recognition of the effector protein AvrBs1 (Minsavage et al., 1990). All strains induced the AvrBs1-specific HR in leaves of ECW-10R plants, indicating that AvrBs1 was efficiently translocated. This suggests that the AvrBs3Δ2 fusion proteins did not interfere with the activity of the T3S system (Figure 1B).

### The N-terminal 30 amino acids of AvrBs3 target the AvrBs3Δ2 reporter for translocation in the absence of the T3S chaperone HpaB

**Figure 2:**
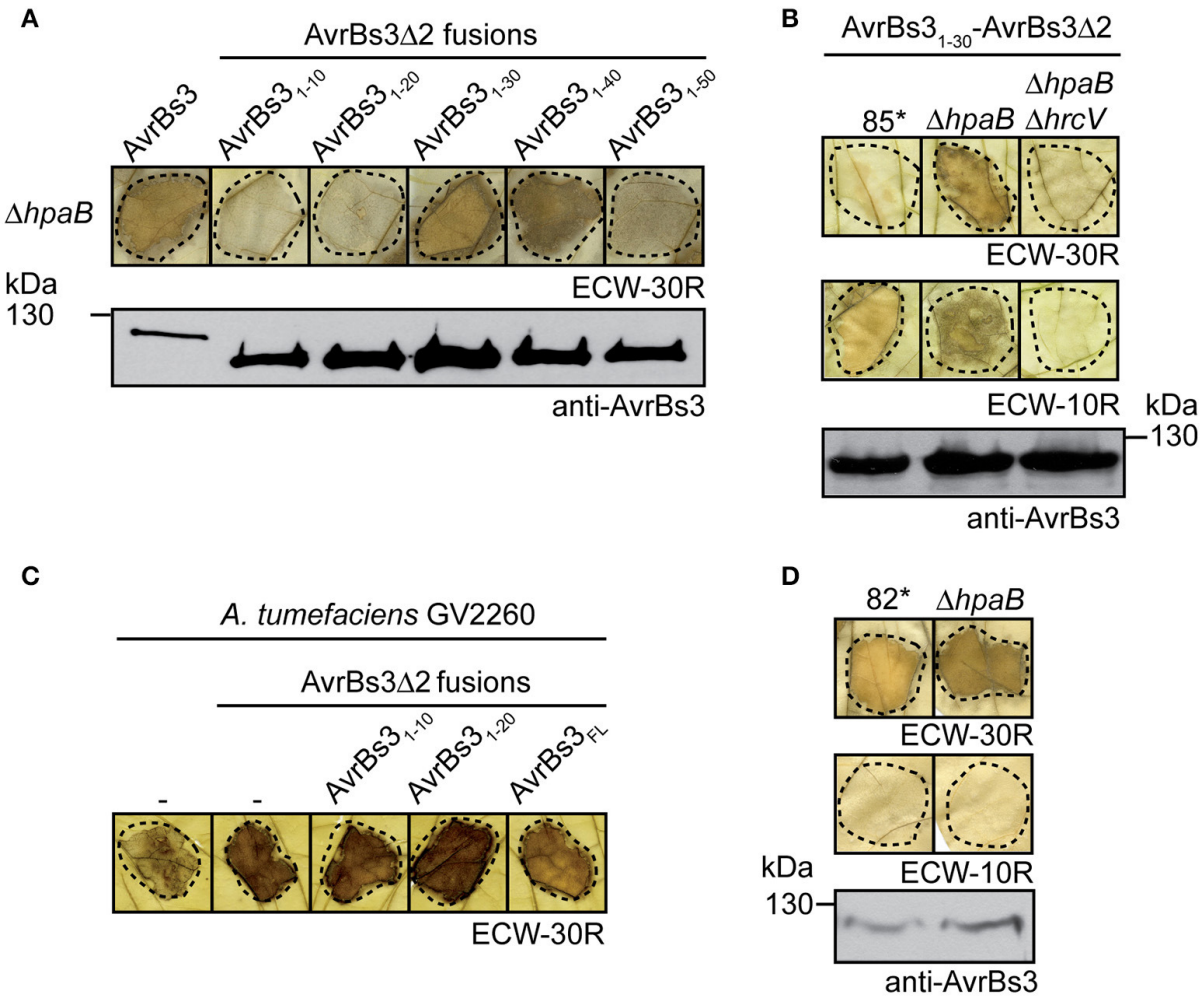
Influence of HpaB on the translocation of AvrBs3 reporter fusions. **(A)** The N-terminal 30 amino acids of AvrBs3 target the AvrBs3Δ2 reporter for translocation in the absence of HpaB. Strain 85^*^Δ*hpaB* (Δ*hpaB*) ectopically expressing *avrBs3* or *avrBs3*Δ*2* fusions as indicated was infiltrated into leaves of AvrBs3-responsive ECW-30R pepper plants. For the better visualization of the HR, leaves were destained in ethanol 3 dpi. Dashed lines indicate the infiltrated areas. Equal amounts of cell extracts were analyzed by immunoblotting using an AvrBs3-specific antiserum. **(B)** Translocation of AvrBs3_1−30_-AvrBs3Δ2 by *hpaB* mutants depends on the T3S system. Strains 85^*^, 85^*^Δ*hpaB* (Δ*hpaB*), and 85^*^Δ*hpaB*Δ*hrcV* (Δ*hpaB*Δ*hrcV*) ectopically expressing *avrBs3*_*1-30*_*-avrBs3*Δ*2* were infiltrated into leaves of AvrBs3-responsive ECW-30R and AvrBs1-responsive ECW-10R pepper plants. Plant reactions and protein synthesis were analyzed as described in **(A)**. The HR in ECW-30R plants was specifically induced by AvrBs3_1−30_-AvrBs3Δ2 and was not observed after infiltration of *X. campestris* pv. *vesicatoria* strains without expression constructs (Figure S1). **(C)** Transient expression of *avrBs3*Δ*2* fusions induces the HR in AvrBs3-responsive pepper plants. *A. tumefaciens* without expression construct (−) or ectopically expressing *avrBs3* or *avrBs3*Δ*2* fusions as indicated was infiltrated at a density of 8 × 10^8^ CFU ml^−1^ into leaves of AvrBs3-responsive pepper plants. Leaves were destained in ethanol 4 dpi. **(D)** AvrBs3 is translocated in the absence of the T3S chaperone HpaB. Strains 82^*^ and 82^*^Δ*hpaB* (Δ*hpaB*) were infiltrated into leaves of AvrBs3-responsive ECW-30R and AvrBs1-responsive ECW-10R pepper plants. Plant reactions and protein synthesis were analyzed as described in **(A)**.

Next, we investigated the influence of the T3S chaperone HpaB on the translocation of AvrBs3Δ2 fusion proteins. Notably, AvrBs3_1−30_-AvrBs3Δ2 was translocated by strain 85^*^Δ*hpaB* but did not induce a macroscopically visible HR in AvrBs3-responsive pepper plants when analyzed in strain 85^*^ or the T3S-deficient strain 85^*^Δ*hpaB*Δ*hrcV* (Figures 2A,B; see above). Similar data were obtained for the *hrpG* wild-type strain 85-10Δ*hpaB*, suggesting that the translocation of AvrBs3_1−30_-AvrBs3Δ2 by *hpaB* deletion mutants was not caused by the overexpression of the T3S genes in the presence of HrpG^*^ (Figure S1). Hence, the N-terminal 30 amino acids of AvrBs3 contain a translocation signal which is recognized in *hpaB* deletion mutants but is not sufficient for efficient translocation in the wild-type strain. This signal will hereafter be referred to as minimal translocation signal. Translocation in the absence of HpaB was also observed for AvrBs3_1−40_-AvrBs3Δ2. In contrast, translocation of AvrBs3_1−50_-AvrBs3Δ2 was significantly reduced in strain 85^*^Δ*hpaB* (Figure 2A) and not detectable in the *hrpG* wild-type strain 85-10Δ*hpaB* (Figure S1), suggesting that the N-terminal 50 amino acids of AvrBs3 depend on HpaB for efficient translocation.

AvrBs3Δ2 fusion proteins containing the N-terminal 10 or 20 amino acids of AvrBs3 did not induce a visible HR when analyzed in strain 85^*^Δ*hpaB* (Figure 2A). To exclude that the N-terminal 10 or 20 amino acids of AvrBs3 interfere with the activity of the AvrBs3Δ2 reporter, both gene fusions were expressed under control of the *35S* promoter in AvrBs3-responsive pepper plants after *A. tumefaciens-*mediated gene delivery. Transient expression of *avrBs3*_*1-10*_- and *avrBs3*_*1-20*_*-avrBs3*Δ*2* led to the induction of the *Bs3*-specific HR, indicating that the AvrBs3Δ2 reporter was functional when present as a fusion partner of the N-terminal 10 or 20 amino acids of AvrBs3 (Figure 2C). Thus, the lack of HR induction by AvrBs3_1−10_- and AvrBs3_1−20_-AvrBs3Δ2 fusion proteins was presumably not caused by a misfolding of the reporter. Taken together, we conclude from these data that the N-terminal 50 amino acids of AvrBs3 contain a translocation signal whereas the N-terminal 30 amino acids promote translocation of AvrBs3 in the absence of HpaB. The N-terminal 10 or 20 amino acids did not target the AvrBs3Δ2 reporter for detectable translocation, however, it cannot be excluded that this region contains a translocation signal which is inactive in the context of the AvrBs3Δ2 fusion.

### Amino acids 64–152 promote translocation of AvrBs3

**Figure 3:**
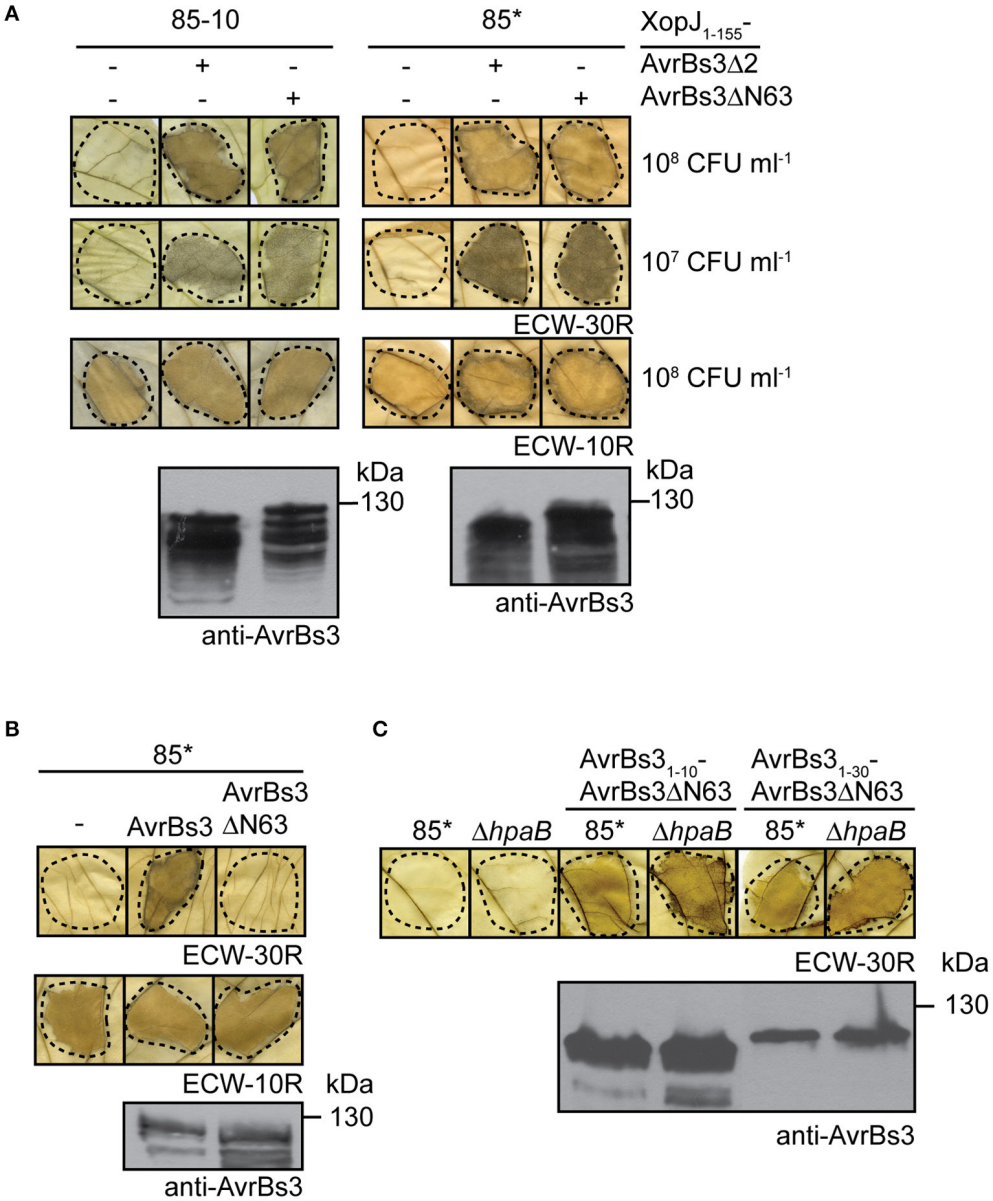
AvrBs3ΔN63 contains a signal that promotes translocation. **(A)** Translocation assays with AvrBs3Δ2 and AvrBs3ΔN63 fusion proteins. Strains 85-10 and 85^*^ without expression construct (−) or ectopically expressing *xopJ*_*1-155*_*-avrBs3*Δ*2* or *xopJ*_*1-155*_*-avrBs3*Δ*N63* were infiltrated at densities of 10^8^ and 10^7^ CFU ml^−1^ into leaves of AvrBs3-responsive ECW-30R and AvrBs1-responsive ECW-10R pepper plants as indicated. Leaves were destained in ethanol 3 dpi. Dashed lines indicate the infiltrated areas. Equal amounts of cell extracts were analyzed by immunoblotting using an AvrBs3-specific antiserum. **(B)** AvrBs3ΔN63 does not induce a macroscopic HR when analyzed in strain 85^*^. Strain 85^*^ without expression construct (−) or ectopically expressing *avrBs3* or *avrBs3*Δ*N63* as indicated was infiltrated into leaves of AvrBs3-responsive ECW-30R and AvrBs1-responsive ECW-10R pepper plants. Plant reactions and protein synthesis were analyzed as described in **(A)**. **(C)** The N-terminal 10 amino acids of AvrBs3 target AvrBs3ΔN63 for translocation in wild-type and *hpaB* mutant strains. Strains 85^*^ and 85^*^Δ*hpaB* (Δ*hpaB*) without expression construct or ectopically expressing *avrBs3*_*1-10*_*-avrBs3*Δ*N63* or *avrBs3*_*1-30*_*-avrBs3*Δ*N63* as indicated were infiltrated at a density of 10^8^ CFU ml^−1^ into leaves of AvrBs3-responsive pepper plants. Leaves were destained in ethanol 2 dpi. Dashed lines indicate the infiltrated areas. Protein synthesis was analyzed as described in **(A)**.

In contrast to AvrBs3_1−50_-AvrBs3Δ2, the full-length AvrBs3 protein induced the HR when analyzed in strains 85^*^Δ*hpaB* and 85-10Δ*hpaB*, suggesting that it was efficiently translocated in the absence of HpaB (Figure 2A; Figure S1). Similar findings were observed for strain 82^*^Δ*hpaB*, which is a derivative of strain 82-8 and naturally expresses *avrBs3* (Figure 2D). This indicates that the HpaB-independent translocation of AvrBs3 was not caused by the overexpression of T3S genes or of *avrBs3*. We, therefore, wondered whether the differences in HR induction by AvrBs3 and AvrBs3_1−50_-AvrBs3Δ2 were caused by differences in protein activities. Previous reporter assays revealed that the N-terminal deletion derivative AvrBs3Δ2 led to reduced activation of an AvrBs3-responsive promoter when compared with the full-length AvrBs3 protein (Schreiber et al., 2015). In contrast, transcription activation by an AvrBs3 derivative, which was deleted in the N-terminal 63 amino acids, was like wild type, suggesting that the region between amino acids 63 and 152 of AvrBs3 contributes to protein activity (Schreiber et al., 2015). To investigate whether different activities of AvrBs3 derivatives result in different HR intensities, we compared the HR induction by AvrBs3Δ2 and AvrBs3Δ63 fusion proteins. As fusion partner, which provides the T3S and translocation signal, we chose the N-terminal 155 amino acids of the effector protein XopJ (Noël et al., 2003). When strain 85^*^ or 85-10 delivering XopJ_1−155_-AvrBs3Δ2 or XopJ_1−155_-AvrBs3Δ63 was infiltrated into leaves of AvrBs3-responsive pepper plants, no differences in the HR intensities were detected, even when bacteria were infiltrated at lower optical densities (Figure 3A). As both fusion proteins were stably synthesized at comparable levels (Figure 3A), we conclude that the previously observed reduced transcription activation activity of AvrBs3Δ2 did not lead to a macroscopically detectable reduction of the AvrBs3-induced HR. The efficient translocation of AvrBs3 by *hpaB* deletion mutants might, therefore, be caused by the presence of additional export signals which promote translocation in the absence of HpaB and are absent in AvrBs3Δ2 fusion proteins.

To investigate the presence of additional export signals outside the N-terminal 50 amino acids of AvrBs3, we analyzed the translocation of fusion proteins between the N-terminal 10 or 30 amino acids of AvrBs3 and AvrBs3Δ63, which lacks the T3S and translocation signal (Schreiber et al., 2015; Scheibner et al., 2016; Figure 3B). Notably, AvrBs3_1−10_- and AvrBs3_1−30_-AvrBs3Δ63 induced the AvrBs3-specific HR when delivered by strains 85^*^ and 85^*^Δ*hpaB* (Figure 3C). This is in contrast to the corresponding AvrBs3Δ2 fusions and suggests that the region between amino acids 64 and 152 of AvrBs3 contains a signal which promotes translocation even in the absence of HpaB.

### AvrBs3 is delivered into plant cells in the absence of the translocon protein HrpF

**Figure 4:**
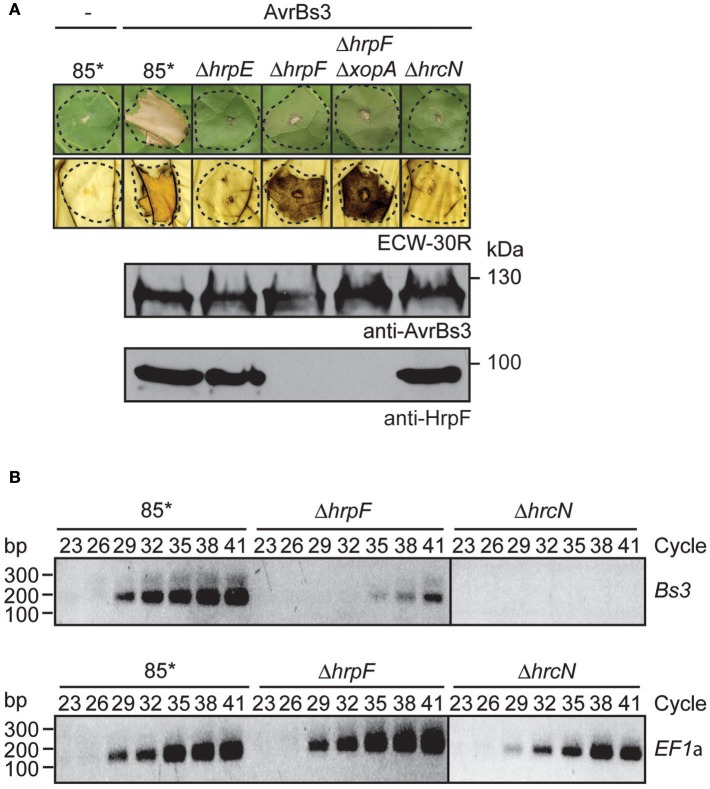
AvrBs3 enters plant cells in the absence of a functional translocon. **(A)** HrpF-independent entry of AvrBs3 into pepper cells. Strains 85^*^, 85^*^Δ*hrpE* (Δ*hrpE*), 85^*^Δ*hrpF* (Δ*hrpF*), 85^*^Δ*hrpF*Δ*xopA* (Δ*hrpF*Δ*xopA*), and 85^*^Δ*hrcN* (Δ*hrcN*) without expression construct (−) or ectopically expressing *avrBs3* as indicated were infiltrated into leaves of AvrBs3-responsive ECW-30R and AvrBs1-responsive ECW-10R pepper plants. Leaves were photographed and destained in ethanol 3 dpi. Dashed lines indicate the infiltrated areas. For the analysis of protein synthesis, equal amounts of cell extracts were analyzed by immunoblotting using AvrBs3- or HrpF-specific antibodies. **(B)** HrpF-independent delivery of AvrBs3 induces the expression of *Bs3* in AvrBs3-responsive pepper plants. Strains 85^*^, 85^*^Δ*hrpF* (Δ*hrpF*), and 85^*^Δ*hrcN* (Δ*hrcN*) ectopically expressing *avrBs3* were infiltrated into leaves of AvrBs3-responsive pepper plants. Eight hours post inoculation, RNA was isolated from infected leaf material and transcribed into cDNA. Fragments corresponding to the *Bs3* transcript and the constitutively expressed gene *EF1*α were amplified for 23–41 PCR cycles as indicated and amplicons were analyzed by agarose gel electrophoresis.

It was previously reported that the translocation of effector proteins from *Xanthomonas* spp. depends on the translocon protein HrpF (Büttner et al., 2002; Szurek et al., 2002; Hotson et al., 2003; Thieme et al., 2007; Jiang et al., 2009; Teper et al., 2016). To confirm the contribution of HrpF to the translocation of AvrBs3, we performed translocation studies with *hrpF* deletion mutants. Unexpectedly, strain 85^*^Δ*hrpF* ectopically expressing *avrBs3* induced a browning of the infected leaf tissue, which was visible after destaining of the leaves in ethanol 3 dpi (Figure 4A). A similar phenotype was observed with strain 85^*^Δ*hrpF*Δ*xopA*, which additionally lacks the secreted XopA protein (Figure 4A; Noël et al., 2002). XopA contributes to pathogenicity and is homologous to harpins, which are proposed to be involved in effector protein translocation (Noël et al., 2002; Kim et al., 2004). No browning of the leaf tissue was visible when *avrBs3* was ectopically expressed in strains 85^*^Δ*hrpE* and 85^*^Δ*hrcN*, which are deleted in the pilus gene *hrpE* and the ATPase gene *hrcN*, respectively (Figure 4A).

To investigate whether the browning of the infected leaf tissue could have resulted from the induction of the *R* gene *Bs3*, we performed RT-PCR studies. The *Bs3* transcript was amplified from leaf tissue infected with strain 85^*^ ectopically expressing *avrBs3* but not from tissue infected with the corresponding *hrcN* deletion mutant (Figure 4B). Reduced amounts of the *Bs3* transcript were detected in leaf material infected with the *hrpF* deletion mutant, suggesting that AvrBs3 entered the plant cells in the absence of the translocon protein HrpF and induced the expression of *Bs3*, albeit in reduced amounts (Figure 4B). We assume that the HrpF-independent entry of AvrBs3 into plant cells was not sufficient for the induction of a macroscopically visible HR reaction in *Bs3* pepper plants but resulted in a browning of the infected leaf tissue. The phenotypes were not caused by the overexpression of *avrBs3* or the T3S genes, because browning of the leaf tissue was also observed after inoculation of *Bs3* pepper plants with the *hrpF* deletion mutant strain 82-8Δ*hrpF*, which contains the *hrpG* wild-type gene and naturally expresses *avrBs3* (Figure S2). In agreement with these observations, the *Bs3* transcript was detectable in leaf material infected with strain 82-8Δ*hrpF* but not with the T3S-deficient strain 82-8Δ*hrcV* (Figure S2).

### Translocon-independent entry of a TAL effector into cells of the non-host plant *N. benthamiana*

**Figure 5:**
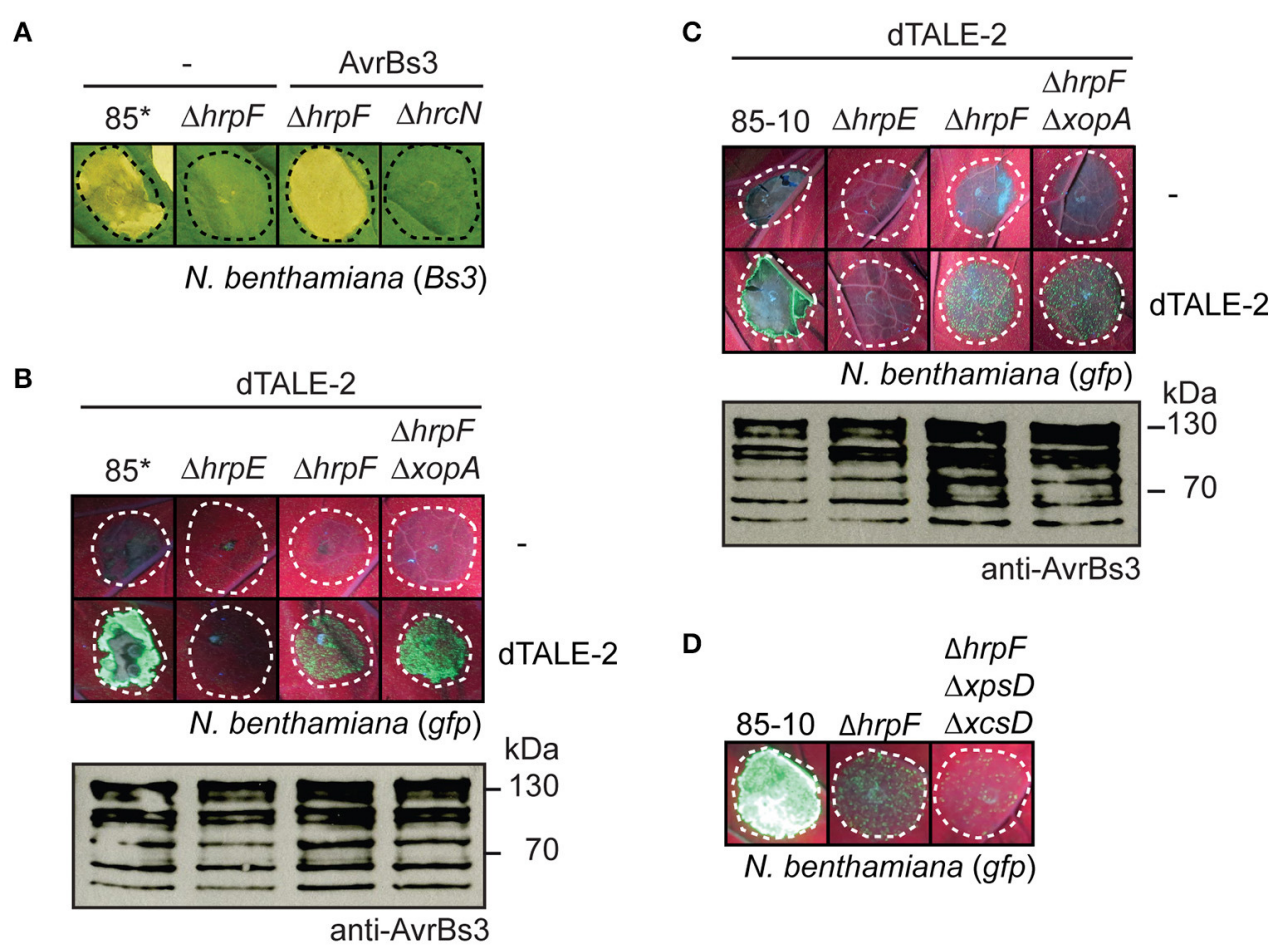
AvrBs3 and dTALE-2 are delivered into *N. benthamiana* in the absence of a functional translocon. **(A)** HrpF-independent entry of AvrBs3 into plant cells induces the HR in *Bs3 N. benthamiana* plants. Strains 85^*^, 85^*^Δ*hrpF* (Δ*hrpF*) and 85^*^Δ*hrcN* (Δ*hrcN*) without expression construct (−) or ectopically expressing *avrBs3* as indicated were infiltrated at a density of 8 × 10^8^ CFU ml^−1^ into leaves of *Bs3*-transgenic *N. benthamiana* plants. Leaves were photographed 8 dpi. Dashed lines indicate the infiltrated areas. **(B)** Translocon-independent delivery of dTALE-2 into *gfp*-transgenic *N. benthamiana* plants. Strains 85^*^, 85^*^Δ*hrpE* (Δ*hrpE*), 85^*^Δ*hrpF* (Δ*hrpF*), and 85^*^Δ*hrpF*Δ*xopA* (Δ*hrpF*Δ*xopA*) without expression construct (−) or ectopically expressing *dTALE-2* as indicated were infiltrated at a density of 5 × 10^8^ CFU ml^−1^ into leaves of *gfp*-transgenic *N. benthamiana* plants. Fluorescence of infected leaf areas was photographed 6 dpi. Dashed lines indicate the infiltrated areas. Fluorescence is reduced in plant tissue infiltrated with strain 85^*^ because of the induction of the non-host HR. For the analysis of protein synthesis, equal amounts of cell extracts were analyzed by immunoblotting using AvrBs3-specific antibodies. **(C)** Analysis of dTALE-2 in derivatives of strain 85-10. Strains 85-10, 85-10Δ*hrpE* (Δ*hrpE*), 85-10Δ*hrpF* (Δ*hrpF*) and 85-10Δ*hrpF*Δ*xopA* (Δ*hrpF*Δ*xopA*) without expression construct (−) or ectopically expressing *dTALE-2* were infiltrated at a density of 5 × 10^8^ CFU ml^−1^ into leaves of *gfp*-transgenic *N. benthamiana* plants. Fluorescence was photographed 13 dpi. Protein synthesis was analyzed as described in **B**. **(D)** HrpF-independent entry of dTALE-2 into plant cells is reduced in the absence of functional T2S systems. Strains 85-10, 85-10Δ*hrpF* (Δ*hrpF*) and 85-10Δ*hrpF*Δ*xpsD*Δ*xcsD* (Δ*hrpF*Δ*xpsD*Δ*xcsD*), which is deficient in the Xps- and Xcs-T2S systems, ectopically expressing *dTALE-2* were infiltrated into leaves of *gfp*-transgenic *N. benthamiana* plants. Fluorescence was photographed 10 dpi.

To investigate whether the translocon-independent entry of AvrBs3 into plant cells also occurs in non-host plants, we performed infection assays with *Bs3*-transgenic *N. benthamiana* plants. As reported previously, strain 85^*^ induced a non-host HR on *N. benthamiana* plants, which is visible as necrotic area (Figure 5A; Metz et al., 2005; Scheibner et al., 2016). No macroscopic HR was observed after infection with the *hrpF* deletion mutant strain 85^*^Δ*hrpF*, suggesting that the induction of the non-host HR in *N. benthamiana* depends on the T3S translocon (Figure 5A). Strain 85^*^Δ*hrpF* ectopically expressing *avrBs3*, however, induced the HR in *Bs3-*transgenic *N. benthamiana* plants, suggesting that AvrBs3 entered the plant cells in the absence of HrpF (Figure 5A). The reaction was dependent on the T3S system because AvrBs3 did not induce a visible HR when analyzed in strain 85^*^Δ*hrcN*, which lacks the T3S ATPase (Figure 5A).

To confirm these results, we performed translocation assays with *X. campestris* pv. *vesicatoria* strains ectopically expressing *dTALE-2* (*designer TAL effector-2*), which encodes an artificial derivative of AvrBs3 with modified RVDs (Weber et al., 2011b). To analyse the translocation of dTALE-2, bacteria were infiltrated into leaves of transgenic *N. benthamiana* plants, which encode the green fluorescent protein (GFP) under control of a dTALE-2-responsive promoter on a stably integrated viral vector construct (Werner et al., 2011; Scheibner et al., 2016). dTALE-2 induced GFP fluorescence when delivered by strain 85^*^ (Figure 5B; Scheibner et al., 2016). Reduced fluorescence was observed when dTALE-2 was analyzed in strains 85^*^Δ*hrpF* and 85^*^Δ*hrpF*Δ*xopA* whereas no fluorescence was detectable after infiltration of the T3S-deficient strain 85^*^Δ*hrpE* (Figure 5B). Similar data were obtained for derivatives of the wild-type strain 85-10, suggesting that the HrpF-independent entry of dTALE-2 into plant cells was not caused by the overexpression of the T3S genes (Figure 5C). Taken together, these data suggest that dTALE-2 can enter the plant cell in a translocon-independent manner.

We also performed infection experiments with strain 85-10Δ*hrpF*Δ*xpsD*Δ*xcsD*, which lacks *hrpF* and functional T2S systems. Type II-secreted cell wall-degrading enzymes were proposed to facilitate T3S pilus assembly and thus presumably contribute to the HrpF-independent passage of AvrBs3 across the plant plasma membrane (Szczesny et al., 2010b). In agreement with this hypothesis, dTALE-2 induced reduced GFP fluorescence when analyzed in strain 85-10Δ*hrpF*Δ*xpsD*Δ*xcsD* (Figure 5D).

### The translocation signal contributes to the HrpF-independent entry of AvrBs3 into plant cells

**Figure 6:**
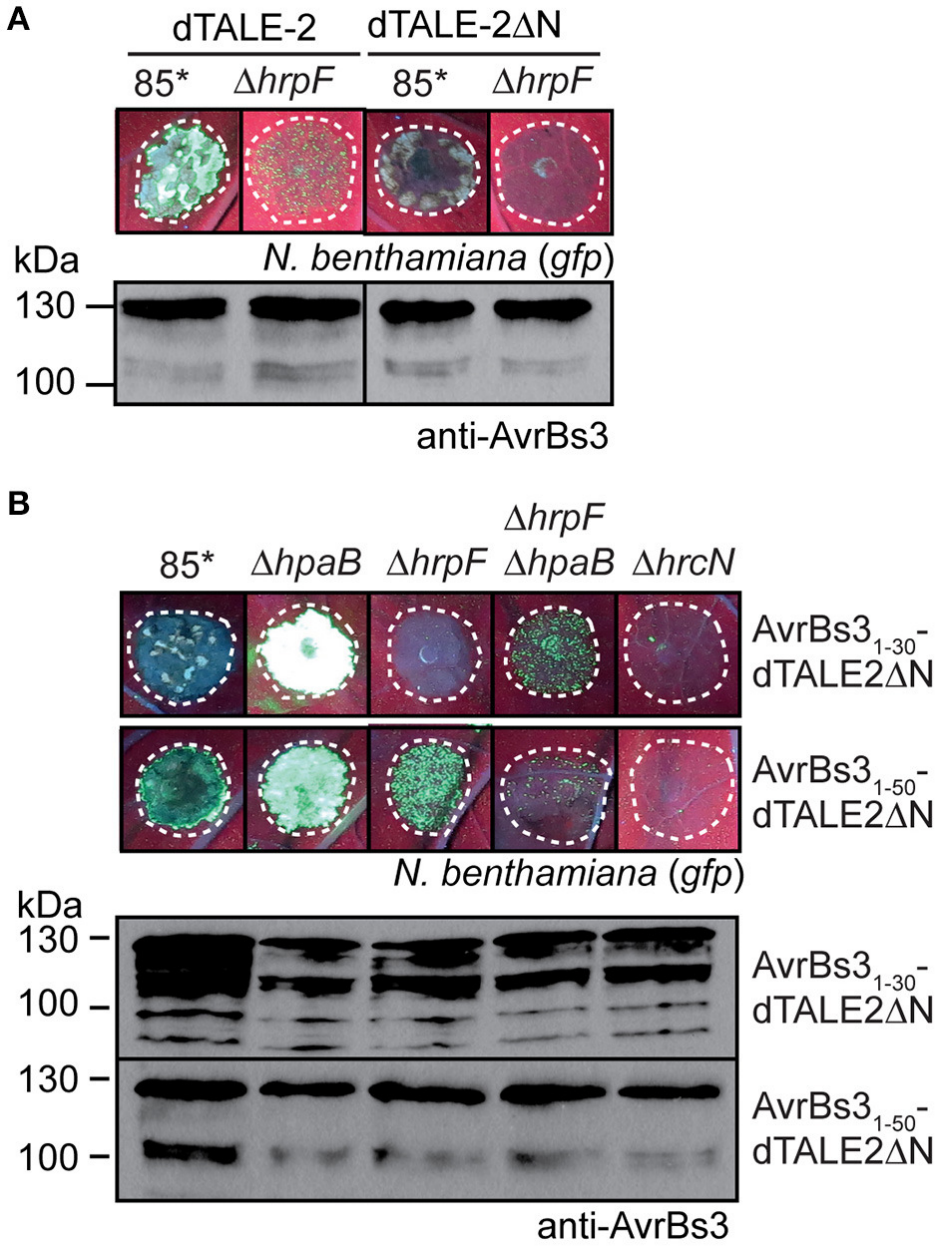
The N-terminal translocation signal is required for translocon-independent delivery of dTALE-2. **(A)** dTALE-2ΔN does not induce detectable GFP fluorescence when analyzed in strain 85^*^. Strains 85^*^ and 85^*^Δ*hrpF* (Δ*hrpF*) ectopically expressing *dTALE-2* or *dTALE-2*Δ*N* as indicated were infiltrated at a density of 8 × 10^8^ CFU ml^−1^ into leaves of *gfp*-transgenic *N. benthamiana* plants. Fluorescence was photographed 7 dpi. Dashed lines indicate the infiltrated areas. For the analysis of protein synthesis, equal amounts of cell extracts were analyzed by immunoblotting using AvrBs3-specific antibodies. **(B)** Translocation assays with dTALE-2ΔN fusion proteins. Strains 85^*^, 85^*^Δ*hpaB* (Δ*hpaB*), 85^*^Δ*hrpF* (Δ*hrpF*), 85^*^Δ*hrpF*Δ*hpaB* (Δ*hrpF*Δ*hpaB*), and 85^*^Δ*hrcN* (Δ*hrcN*) ectopically expressing *dTALE-2*Δ*N* fusions as indicated were infiltrated at a density of 8 × 10^8^ CFU ml^−1^ into leaves of *gfp*-transgenic *N. benthamiana* plants. Fluorescence was photographed 11 dpi. Dashed lines indicate the infiltrated areas. Fluorescence is reduced in plant tissue infiltrated with strain 85^*^ because of the induction of the non-host HR. Protein synthesis was analyzed as described in **(A)**.

Next, we investigated the contribution of the translocation signal to the translocon-independent entry of AvrBs3 into plant cells. Infection experiments showed that the N-terminal deletion derivative dTALE-2ΔN did not induce detectable GFP fluorescence in *gfp-*transgenic *N. benthamiana* plants when analyzed in strains 85^*^ and 85^*^Δ*hrpF* (Figure 6A). As dTALE-2ΔN is deleted in the T3S and translocation signal (Schreiber et al., 2015; Scheibner et al., 2016), the lack of GFP fluorescence suggests that the translocon-independent delivery of dTALE-2 depends on its passage through the T3S system. In contrast to dTALE-2ΔN, AvrBs3_1−50_-dTALE-2ΔN induced GFP fluorescence when analyzed in strains 85^*^, 85^*^Δ*hpaB* and 85^*^Δ*hrpF* (Figure 6B). Notably, GFP fluorescence induced by strain 85^*^ was reduced because of the induction of the non-host HR by strain 85^*^ which leads to tissue necrosis (see above; Figure 5A). Our observations confirm the results obtained for the AvrBs3_1−50_-AvrBs3Δ2 fusion (see above) and suggest that the N-terminal 50 amino acids of AvrBs3 contain a translocation signal, which can target dTALE-2 for translocon-independent translocation.

Translocation assays with AvrBs3_1−30_-dTALE-2ΔN revealed that the N-terminal 30 amino acids of AvrBs3 targeted dTALE-2ΔN for translocation in strain 85^*^Δ*hpaB* whereas no fluorescence was observed with the wild-type strain or the *hrpF* deletion mutant (Figure 6B). This confirms the finding that the N-terminal 30 amino acids of AvrBs3 contain a minimal translocation signal, which is recognized in the absence of HpaB. Reduced GFP fluorescence, however, was detected when AvrBs3_1−30_-dTALE-2ΔN was analyzed in strain 85^*^Δ*hrpF*Δ*hpaB* (Figure 6B). Thus, AvrBs3_1−30_-dTALE-2ΔN can enter the plant cell in the absence of the translocon when delivered by the *hpaB* deletion mutant (Figure 6B). Taken together, these data suggest that the translocon-dependent and -independent delivery of AvrBs3 into plant cells depends on similar targeting signals.

## Discussion

In the present study, we localized T3S and translocation signals in the N-terminal region of the TAL effector AvrBs3. The analysis of AvrBs3-reporter fusion proteins revealed that the N-terminal 10 amino acids of AvrBs3 are sufficient to target the reporter for T3S (Figure 1). This is in agreement with previous findings that amino acids 6–10 of T3S substrates often contain essential features of T3S signals as was also shown for the effector proteins XopE2 and XopJ from *X. campestris* pv. *vesicatoria* (Wang et al., 2013; Scheibner et al., 2017). The N-terminal 30 amino acids of AvrBs3 contain a minimal translocation signal, which promotes translocation of the reporter in the absence of the T3S chaperone HpaB but not in the wild-type strain (Figures 1, 2). Minimal translocation signals were previously also identified in the N-terminal regions of the effectors XopE2 and XopJ (Scheibner et al., 2017).

**Figure 7:**
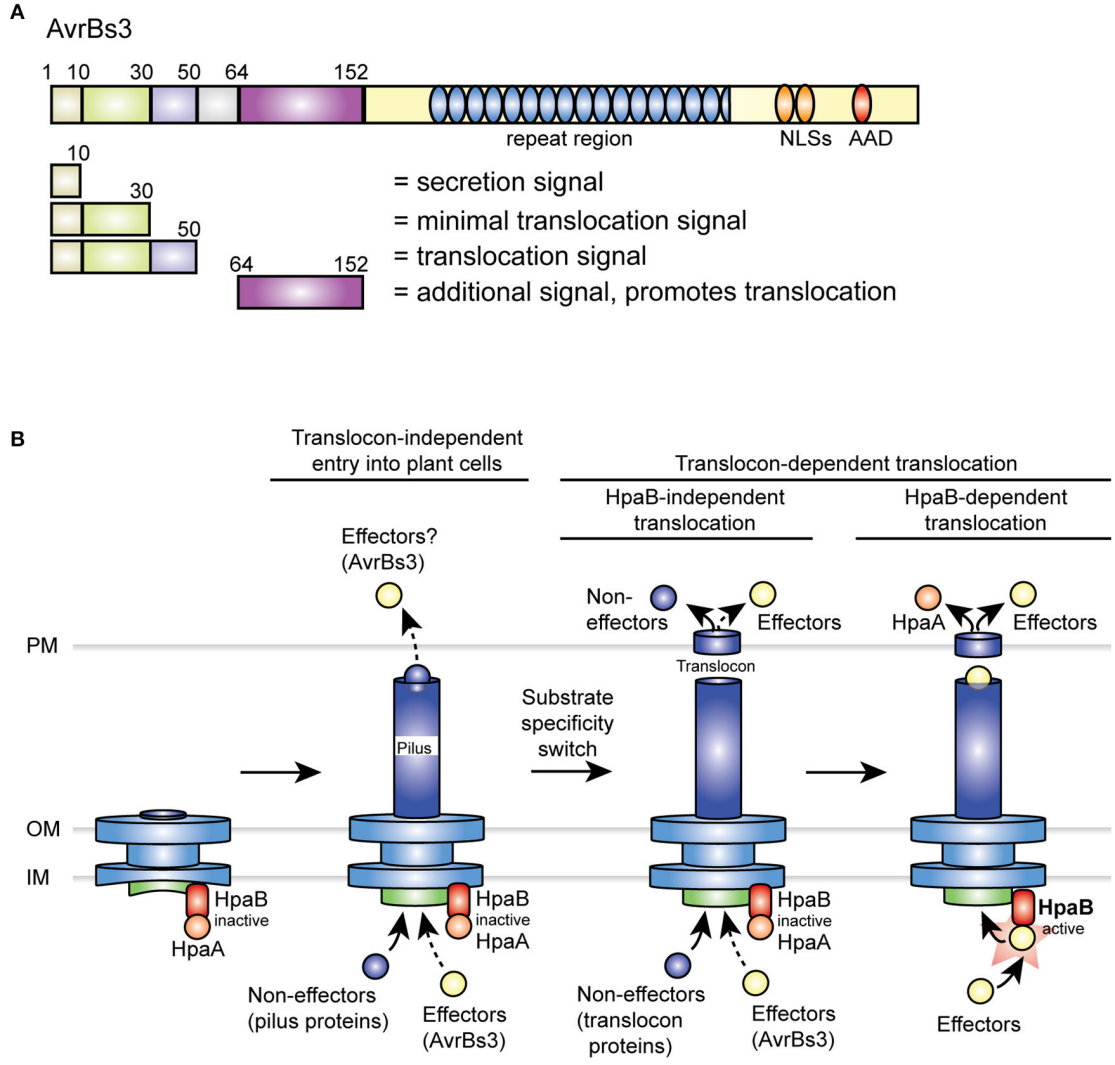
Overview on type III export signals in AvrBs3 and model of the T3S hierarchy in *X. campestris* pv. *vesicatoria*. **(A)** Overview on secretion and translocation signals in AvrBs3. N-terminal signals are indicated, numbers refer to amino acid positions. NLS, nuclear localization signal; AAD, acidic activation domain. **(B)** Predicted T3S hierarchy in *X. campestris* pv. *vesicatoria*. After formation of the membrane-spanning part of the secretion apparatus, the T3S pilus is assembled. HpaA presumably associates with the T3S chaperone HpaB and thus inactivates HpaB to prevent secretion of effector proteins prior to the insertion of the translocon. As suggested by data in the present study, AvrBs3 might already enter the plant cell in the absence of a functional translocon (indicated by a dashed arrow). A possible translocon-independent entry of other effectors into the plant cell (indicated by a question mark) remains to be investigated. A predicted switch in T3S substrate specificity after pilus formation leads to the secretion of translocon proteins and thus to the assembly of the T3S translocon in the plant plasma membrane. While HpaB is still inactive during this stage of the T3S process, HpaB-independent effectors including AvrBs3 and non-effectors such as XopA and HrpF are already translocated. However, when compared to the wild-type strain, *hpaB* mutants presumably translocate reduced amounts of effectors (indicated by a dashed arrow; Büttner et al., 2006; Schulze et al., 2012). After insertion of the translocon, a yet unknown signal triggers the translocation of HpaA. This leads to the liberation of HpaB and thus activates the translocation of HpaB-dependent effectors. AAD, acidic activation domain; IM, inner membrane; NLS, nuclear localization signal; OM, outer membrane; PM, plant plasma membrane.

Translocation of AvrBs3 in the wild-type strain depends on a signal in the N-terminal 50 amino acids. Notably, however, in contrast to the minimal translocation signal, the N-terminal 50 amino acids did not efficiently target the AvrBs3Δ2 reporter for translocation in the absence of HpaB (Figure 2), suggesting that the function of the minimal translocation signal depends on the neighboring protein regions. In contrast to AvrBs3_1−50_-AvrBs3Δ2, however, the full-length AvrBs3 protein was efficiently translocated by both wild-type and *hpaB* mutant strains (Figures 1, 2). AvrBs3 likely contains additional signals outside the N-terminal region, which promote translocation in the absence of HpaB. Thus, the comparative analysis of AvrBs3Δ2 and AvrBs3ΔN63 fusion proteins revealed that the region between amino acids 64 and 152 promotes translocation of AvrBs3 in both wild-type and *hpaB* deletion mutant strains (Figures 3, 7A), suggesting that AvrBs3 contains multiple signals that control secretion and translocation in the presence and absence of HpaB. HpaB-independent translocation was already previously observed for other effectors and is indicative of a hierarchy in effector protein delivery (Büttner et al., 2006; Schulze et al., 2012). Thus, in *X. campestris* pv. *vesicatoria*, HpaB-independent effectors might be translocated during the early stages of the plant-pathogen interaction, prior to the activation of HpaB (Figure 7B). Differences in the timing of effector protein delivery were previously reported for animal-pathogenic bacteria but have not yet been investigated in plant-pathogenic bacteria (Enninga et al., 2005; Schlumberger et al., 2005; Mills et al., 2008; Van Engelenburg and Palmer, 2008; Winnen et al., 2008). It is also still unknown whether the timing of effector protein translocation is controlled by the N-terminal export signals.

Comparative sequence analysis revealed that the N-terminal region of AvrBs3 does not share homology with corresponding regions of other effector proteins, indicating that T3S and translocation signals are not conserved in T3S substrates from *X. campestris* pv. *vesicatoria*. Similar findings were previously reported for T3S substrates from other plant- and animal-pathogenic bacteria (Schechter et al., 2004; Arnold et al., 2009; Löwer and Schneider, 2009; Samudrala et al., 2009; Buchko et al., 2010; McDermott et al., 2011). The analysis of the amino acid composition of AvrBs3 revealed that the N-terminal 50 amino acids of AvrBs3 contain slightly higher levels of arginine and aspartate residues than the remainder of the protein (Figure S3). Furthermore, the N-terminal 50 amino acids of AvrBs3 and the region between amino acids 64 and 152, which both promote translocation, contain increased amounts of proline residues (Figure S3). Notably, elevated proline levels are also present in the N-terminal regions of the effectors XopE2 and XopJ, which contain T3S and minimal translocation signals (Scheibner et al., 2017). The contribution of N-terminal proline, arginine and aspartate residues to the translocation of AvrBs3 remains to be investigated in future studies.

The precise roles of N-terminal T3S and translocation signals as well as the molecular mechanisms underlying their recognition are still largely unknown in both plant- and animal-pathogenic bacteria. Potential docking sites for type III effectors in *X. campestris* pv. *vesicatoria* include the cytoplasmic putative C ring protein HrcQ and the cytoplasmic domain of the inner membrane protein HrcV, which both interact with T3S substrates *in vitro* (Lorenz et al., 2012; Hartmann and Büttner, 2013). In case of AvrBs3, however, the N-terminal protein region, which contains the T3S and translocation signal, is dispensable for the interaction with HrcQ and the cytoplasmic domain of HrcV (Lorenz et al., 2012; Hartmann and Büttner, 2013). Similar findings were observed for N-terminal deletion derivatives of XopE2 and XopJ, which interact with HrcQ (Scheibner et al., 2017). The specific recognition of the N-terminal export signal might, therefore, depend on additional components of the T3S system or might occur after the initial docking of T3S substrates to the secretion apparatus.

Given the anticipated essential role of the translocon for type III-dependent protein translocation, it was assumed that the insertion of the translocon precedes effector protein translocation. In agreement with this hypothesis, previous studies suggested that the translocation of effector-reporter fusions as well as the *in situ* detection of AvrBs3 in plant nuclei is abolished in HrpF-deficient strains (Büttner et al., 2002; Szurek et al., 2002; Hotson et al., 2003; Thieme et al., 2007; Jiang et al., 2009; Teper et al., 2016). In the present study, however, we observed that AvrBs3 enters plant cells even in the absence of the translocon protein HrpF and the secreted XopA protein, which might contribute to effector protein translocation (Figure 4; Noël et al., 2002). Translocon-independent entry of AvrBs3 into plant cells was significantly reduced when compared to the translocation by the wild-type strain and only led to a browning of the infected leaf tissue in *Bs3* pepper plants, suggesting a much weaker HR. This was accompanied by a detectable increase in the *Bs3* transcript level (Figure 4) and has not yet been investigated for the wild-type AvrBs3 protein in previous studies. In *Bs3-*transgenic *N. benthamiana* plants, AvrBs3 induces a macroscopically visible *Bs3*-dependent HR even when delivered by *hrpF* deletion mutants (Figure 5). This was not observed in *Bs3* pepper plants and might be caused by an increased activation of *Bs3* expression, an increased HR induction or a more efficient translocon-independent entry of AvrBs3 in *Bs3-*transgenic *N. benthamiana* plants. HrpF-independent entry into plant cells was confirmed for dTALE-2 in *gfp-*transgenic *N. benthamiana* plants. No detectable fluorescence was observed with mutants lacking the T3S ATPase HrcN or the pilus protein HrpE, suggesting that the HrpF-independent delivery of AvrBs3 depends on the T3S system (Figure 5). Given that the transport of AvrBs3 through the T3S pilus is essential for its passage across the plant cell wall, we did not investigate a possible autonomous entry of recombinant AvrBs3 into plant cells.

The mechanisms underlying the HrpF-independent passage of AvrBs3 into plant cells as well as its biological significance remain to be elucidated. Notably, translocon-independent transport into eukaryotic cells was previously reported for effector proteins from animal-pathogenic bacteria including SspH1 from *Salmonella* spp. and YopM from *Yersinia* spp. (Rüter et al., 2010; Scharnert et al., 2013; Lubos et al., 2014). Both proteins cross the host plasma membrane when added as recombinant proteins to cultured cells. Autonomously translocating effectors present a novel class of cell-penetrating peptides (CPPs), which are often used as vehicles for the transport of cargo molecules into target cells (Rüter and Schmidt, 2017). CPPs include basic/amphiphilic or hydrophobic peptides which consist of usually less than 30 amino acids and autonomously translocate across biological membranes, even when present as fusion partner of a large cargo protein (Takeuchi and Futaki, 2016; Radis-Baptista et al., 2017). CPPs are either directly transported across the membrane or are delivered by endocytosis (Scharnert et al., 2013; Radis-Baptista et al., 2017). Both transport pathways were observed for YopM from *Yersinia* spp. (Scharnert et al., 2013). Our preliminary infection experiments with *X. campestris* pv. *vesicatoria* translocon mutants in the presence of the endocytosis inhibitors bafilomycin A1, cytochalasin D, or wortmannin did not reveal a contribution of endocytosis to the delivery of AvrBs3. It, therefore, remains to be investigated whether AvrBs3 can directly cross biological membranes. The direct transport of CPPs across the membrane presumably involves the interaction of positively charged amino acids with negatively charged lipids of the membrane and was for instance reported for arginine-rich CPPs (Scharnert et al., 2013; Rüter and Schmidt, 2017). Interestingly, the N-terminal region of AvrBs3 is enriched in arginine residues (Figure S3), however, AvrBs3 does not contain sequence motifs with homology to typical CPP sequences or prolonged stretches of positively charged amino acids, which could mediate the interaction with membranes (Radis-Baptista et al., 2017). In future studies, we will investigate which protein regions of AvrBs3 are required for the translocon-independent entry into plant cells.

## Author contributions

FS, DB, and SM conceived the study. FS performed secretion assays, infection studies, immunoblot analyses and RT-PCR experiments. DB performed infection assays and immunoblot analyses. All authors analyzed the data. DB and FS wrote the manuscript. All authors read and approved the final manuscript.

### Conflict of interest statement

The authors declare that the research was conducted in the absence of any commercial or financial relationships that could be construed as a potential conflict of interest.

## Supplementary material

Figure S1 Translocation of AvrBs3Δ2 and AvrBs3ΔN63 fusion proteins by derivatives of strain 85-10. **(A)** The HR induction in ECW-30R pepper plants is specific for AvrBs3Δ2 fusion proteins. Strains 85^*^ and 85^*^Δ*hpaB* (Δ*hpaB*) without expression construct or ectopically expressing *avrBs3*_*1-30*_*-avrBs3*Δ*2* were infiltrated into leaves of AvrBs3-responsive ECW-30R and AvrBs1-responsive ECW-10R pepper plants. For the better visualization of the HR, leaves of ECW-10R and ECW-30R plants were destained in ethanol 2 and 3 dpi, respectively. Dashed lines indicate the infiltrated areas. **(B)** Translocation assays with strains 85-10 and 85-10Δ*hpaB*. Strains 85-10 and 85-10Δ*hpaB* without expression construct (−) or encoding AvrBs3, AvrBs3_1−X_-AvrBs3Δ2 or AvrBs3_1−X_-AvrBs3ΔN63 fusion proteins (1-X = amino acids 1–30 or 1–50 of AvrBs3) on corresponding expression constructs as indicated were infiltrated into leaves of AvrBs3-responsive ECW-30R and AvrBs1-responsive ECW-10R pepper plants. Plant reactions were analyzed as described in **(A)**. **(C)** Detection of AvrBs3Δ2 and AvrBs3ΔN63 fusion proteins. Equal amounts of cell extracts from strains described in **(B)** were analyzed by immunoblotting using an AvrBs3-specific antiserum.

Figure S2 Translocon-independent delivery of the native AvrBs3 protein. **(A)** Infection assays with derivatives of strain 82-8. Strains 82-8, 82-8Δ*hrpF* (Δ*hrpF*), 82-8Δ*hrpF*Δ*xopA* (Δ*hrpF*Δ*xopA*), and 82-8Δ*hrcV* (Δ*hrcV*) were infiltrated at a density of 8 × 10^8^ CFU ml^−1^ into leaves of AvrBs3-responsive ECW-30R pepper plants and *Bs3-*transgenic *N. benthamiana* plants. Leaves were destained in ethanol 3 and 5 dpi, respectively. Dashed lines indicate the infiltrated areas. Equal amounts of cell extracts were analysed by immunoblotting using an AvrBs3-specific antiserum. **(B)** Analysis of *Bs3* transcript levels. Strains 82-8, 82-8Δ*hrpF* (Δ*hrpF*), 82-8Δ*hrpF*Δ*xopA* (Δ*hrpF*Δ*xopA*), and 82-8Δ*hrcV* (Δ*hrcV*) were infiltrated at a density of 8 × 10^8^ CFU ml^−1^ into leaves AvrBs3-responsive ECW-30R pepper plants and *Bs3-*transgenic *N. benthamiana* plants. RNA was isolated from infected leaf material and transcribed into cDNA. Fragments corresponding to *Bs3* and the constitutively expressed *EF1*α gene were amplified for 40 cycles by PCR and the amplification products were analyzed by agarose gel electrophoresis.

Figure S3 Amino acid composition of AvrBs3. The percentage of proline, polar and charged amino acid residues in different regions of AvrBs3 is shown.
