# Supplementary figures and images for: The TAL Effector AvrBs3 from Xanthomonas campestris pv. vesicatoria Contains Multiple Export Signals and Can Enter Plant Cells in the Absence of the Type III Secretion Translocon
Source: Front Microbiol. 2017 Nov 9;8:2180. doi: 10.3389/fmicb.2017.02180 (PMC5684485; doi:10.3389/fmicb.2017.02180)

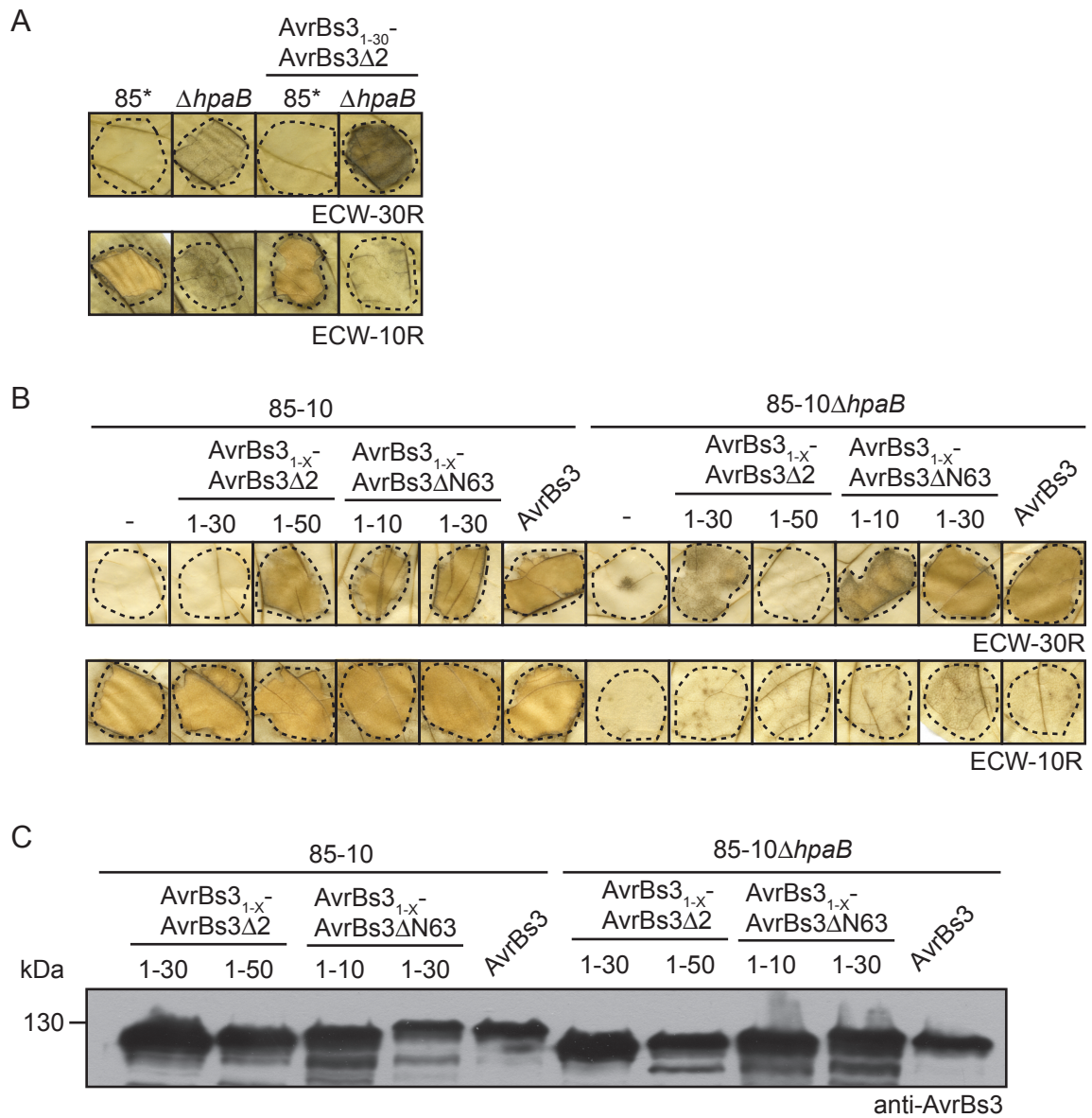

Supplement: Figure S1 — Translocation of AvrBs3Δ2 and AvrBs3ΔN63 fusion proteins by derivatives of strain 85-10. (A) The HR induction in ECW-30R pepper plants is specific for AvrBs3Δ2 fusion proteins. Strains 85* and 85*ΔhpaB (ΔhpaB) without expression construct or ectopically expressing avrBs31-30-avrBs3Δ2 were infiltrated into leaves of AvrBs3-responsive ECW-30R and AvrBs1-responsive ECW-10R pepper plants. For the better visualization of the HR, leaves of ECW-10R and ECW-30R plants were destained in ethanol 2 and 3 dpi, respectively. Dashed lines indicate the infiltrated areas. (B) Translocation assays with strains 85-10 and 85-10ΔhpaB. Strains 85-10 and 85-10ΔhpaB without expression construct (−) or encoding AvrBs3, AvrBs31−X-AvrBs3Δ2 or AvrBs31−X-AvrBs3ΔN63 fusion proteins (1-X = amino acids 1–30 or 1–50 of AvrBs3) on corresponding expression constructs as indicated were infiltrated into leaves of AvrBs3-responsive ECW-30R and AvrBs1-responsive ECW-10R pepper plants. Plant reactions were analyzed as described in (A). (C) Detection of AvrBs3Δ2 and AvrBs3ΔN63 fusion proteins. Equal amounts of cell extracts from strains described in (B) were analyzed by immunoblotting using an AvrBs3-specific antiserum. [file Image1.PDF]
