# Supplementary material for: The TAL Effector AvrBs3 from Xanthomonas campestris pv. vesicatoria Contains Multiple Export Signals and Can Enter Plant Cells in the Absence of the Type III Secretion Translocon
Source: Front Microbiol. 2017 Nov 9;8:2180. doi: 10.3389/fmicb.2017.02180 (PMC5684485; doi:10.3389/fmicb.2017.02180)
Supplement: Figure S2 — Translocon-independent delivery of the native AvrBs3 protein. (A) Infection assays with derivatives of strain 82-8. Strains 82-8, 82-8ΔhrpF (ΔhrpF), 82-8ΔhrpFΔxopA (ΔhrpFΔxopA), and 82-8ΔhrcV (ΔhrcV) were infiltrated at a density of 8 × 10^8^ CFU ml−1 into leaves of AvrBs3-responsive ECW-30R pepper plants and Bs3-transgenic N. benthamiana plants. Leaves were destained in ethanol 3 and 5 dpi, respectively. Dashed lines indicate the infiltrated areas. Equal amounts of cell extracts were analysed by immunoblotting using an AvrBs3-specific antiserum. (B) Analysis of Bs3 transcript levels. Strains 82-8, 82-8ΔhrpF (ΔhrpF), 82-8ΔhrpFΔxopA (ΔhrpFΔxopA), and 82-8ΔhrcV (ΔhrcV) were infiltrated at a density of 8 × 10^8^ CFU ml−1 into leaves AvrBs3-responsive ECW-30R pepper plants and Bs3-transgenic N. benthamiana plants. RNA was isolated from infected leaf material and transcribed into cDNA. Fragments corresponding to Bs3 and the constitutively expressed EF1α gene were amplified for 40 cycles by PCR and the amplification products were analyzed by agarose gel electrophoresis. [file Image2.PDF]

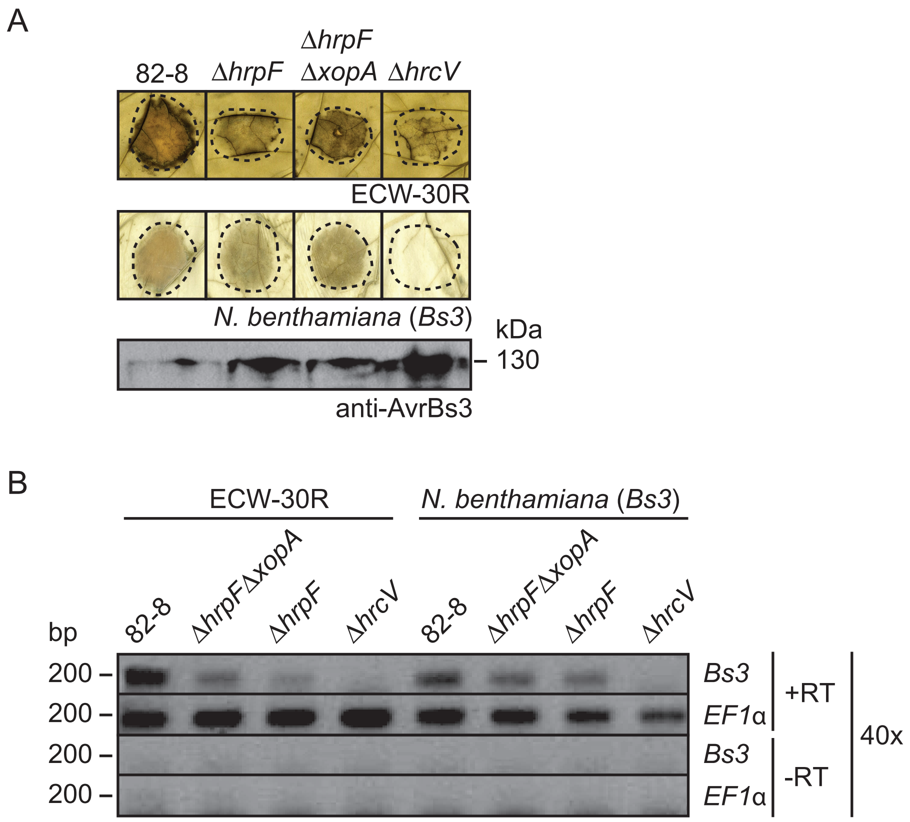

Scheibner et al.
Figure S2

Fig. S2 Translocon-independent delivery of the native AvrBs3 protein.

(A) Infection assays with derivatives of strain 82-8. Strains 82-8, 82-8Δ*hrpF* (Δ*hrpF*), 82-8Δ*hrpF*Δ*xopA* (Δ*hrpF*Δ*xopA*) and 82-8Δ*hrcV* (Δ*hrcV*) were infiltrated at a density of 8 × 10^8 CFU ml^{-1} into leaves of AvrBs3-responsive ECW-30R pepper plants and *Bs3*-transgenic *N. benthamiana* plants. Leaves were destained in ethanol 3 and 5 dpi, respectively. Dashed lines indicate the infiltrated areas. Equal amounts of cell extracts were analysed by immunoblotting using an AvrBs3-specific antiserum. **(B)** Analysis of *Bs3* transcript levels. Strains 82-8, 82-8Δ*hrpF* (Δ*hrpF*), 82-8Δ*hrpF*Δ*xopA* (Δ*hrpF*Δ*xopA*) and 82-8Δ*hrcV* (Δ*hrcV*) were infiltrated at a density of 8 × 10^8 CFU ml^{-1} into leaves AvrBs3-responsive ECW-30R pepper plants and *Bs3*-transgenic *N. benthamiana* plants. RNA was isolated from infected leaf material and transcribed into cDNA. Fragments corresponding to *Bs3* and the constitutively expressed *EF1α* gene were amplified for 40 cycles by PCR and the amplification products were analysed by agarose gel electrophoresis.
