# Supplementary material for: The TAL Effector AvrBs3 from Xanthomonas campestris pv. vesicatoria Contains Multiple Export Signals and Can Enter Plant Cells in the Absence of the Type III Secretion Translocon
Source: Front Microbiol. 2017 Nov 9;8:2180. doi: 10.3389/fmicb.2017.02180 (PMC5684485; doi:10.3389/fmicb.2017.02180)
Supplement: Figure S3 — Amino acid composition of AvrBs3. The percentage of proline, polar and charged amino acid residues in different regions of AvrBs3 is shown. [file Image3.PDF]

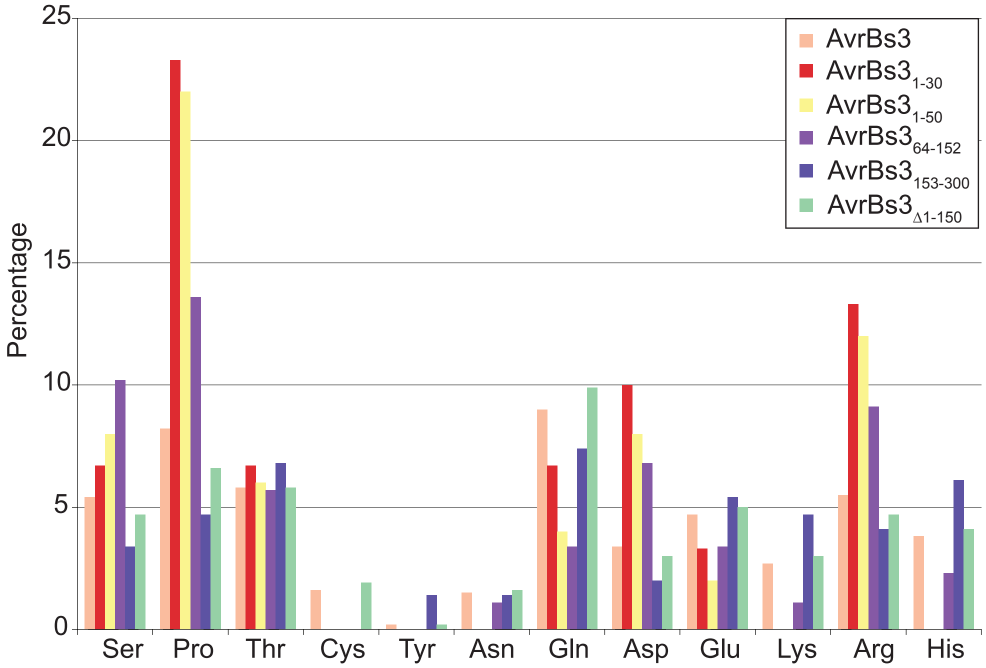

Scheibner et al.
Figure S3

Fig. S3 Amino acid composition of AvrBs3.

The percentage of proline, polar and charged amino acid residues in different regions of AvrBs3 is shown.
